# A complex of the lipid transport ER proteins TMEM24 and C2CD2 with band 4.1 at cell–cell contacts

**DOI:** 10.1083/jcb.202311137

**Published:** 2024-08-19

**Authors:** Ben Johnson, Maria Iuliano, TuKiet T. Lam, Thomas Biederer, Pietro V. De Camilli

**Affiliations:** 1 Department of Neuroscience, Yale University School of Medicine, New Haven, CT, USA; 2 Department of Cell Biology, Yale University School of Medicine, New Haven, CT, USA; 3 Department of Neurology, Yale University School of Medicine, New Haven, CT, USA; 4 Department of Molecular Biophysics and Biochemistry, Yale University School of Medicine, New Haven, CT, USA; 5 Program in Cellular Neuroscience, Neurodegeneration and Repair, Yale University School of Medicine, New Haven, CT, USA; 6 Howard Hughes Medical Institute, Yale University School of Medicine, New Haven, CT, USA; 7 Department of Keck MS and Proteomics Resource, Yale University School of Medicine, New Haven, CT, USA; 8 Department of Neuroscience, Tufts University School of Medicine, Boston, MA, USA

## Abstract

Junctions between the ER and plasma membrane (PM) are implicated in calcium homeostasis, non-vesicular lipid transfer, and other cellular functions. Two ER proteins that function both as tethers to the PM via a polybasic C-terminus motif and as phospholipid transporters are brain-enriched TMEM24 (C2CD2L) and its paralog C2CD2. We report that both proteins also form a complex with band 4.1 family members, which in turn bind PM proteins including cell adhesion molecules such as SynCAM 1. This complex enriches TMEM24 and C2CD2 containing ER/PM junctions at sites of cell contacts. Dynamic properties of TMEM24-dependent ER/PM junctions are impacted when band 4.1 is part of the junction, as TMEM24 at cell-adjacent ER/PM junctions is not shed from the PM by calcium rise, unlike TMEM24 at non-cell adjacent junctions. Lipid transport between the ER and the PM by TMEM24 and C2CD2 at sites where cells, including neurons, contact other cells may participate in adaptive responses to cell contact-dependent signaling.

## Teaser

The ER-anchored lipid transfer protein TMEM24/C2CD2L and its paralog C2CD2 mediate the formation of ER–plasma membrane junctions at sites of cell–cell contacts by interacting with band 4.1 family members and indirectly with cell adhesion proteins.

## Introduction

Junctions between the endoplasmic reticulum and the plasma membrane (ER/PM junctions) are sites where the ER is held in close apposition (∼15–25 nm distance) to the PM by protein tethers without undergoing fusion (Chung et al., 2022; Elbaz and Schuldiner, 2011; Fernández-Busnadiego et al., 2015; Rosenbluth, 1962; Wu et al., 2018). Most ER/PM tethers have additional roles beyond holding the two membranes together (Balla et al., 2020; Chen et al., 2019; Gatta and Levine, 2017; Johnson et al., 2018; Kirmiz et al., 2018; Saheki and De Camilli, 2017; Scorrano et al., 2019; Wu et al., 2018). The abundance of ER/PM junctions varies from cell type to cell type and, within a given cell type, they can be heterogeneous in molecular composition and morphology, such as the area of membrane apposition and width of the ER lumen. Their abundance and structure can also differ on PM surfaces that face different neighbors (Chang et al., 2017; Chung et al., 2022; Deardorff et al., 2014; Wu et al., 2017). Moreover, ER/PM junctions can be populated by different proteins and undergo expansion and reduction in size depending on the functional state of the cell (Chang et al., 2017; Giordano et al., 2013). In neurons, where ER/PM junctions can cover as much as 10% of the PM (Kuijpers et al., 2023; Wu et al., 2017) such junctions can decrease in both size and number by ∼50% during depolarization with high K^+^ or NMDA treatment with differences in sensitivity being linked to junction morphology (Tao-Cheng, 2018).

Proteins that both localize at ER/PM junctions and also participate in their formation include ion channels such as Kv2 potassium channels in neurons (Fox et al., 2015; Johnson et al., 2018; Kirmiz et al., 2018), the ER protein STIM1, which plays a critical role in the regulation of store-operated Ca^2+^ entry via its binding the PM Ca^2+^ channel Orai (Chang et al., 2017), as well as a multiplicity of proteins implicated in the non-vesicular transport of lipids (Amos et al., 2023; Chang et al., 2017; Chang et al., 2013; Chung et al., 2023; Johnson et al., 2019; Saheki and De Camilli, 2017; Suzuki et al., 2014; Thakur et al., 2019; Kim et al., 2015). This mode of lipid transport is critically important as most lipid species in eukaryotic cells are synthesized within the ER. Thus, homeostasis of PM lipids, which is needed to support the structural and signaling functions of this membrane, requires rapid and efficient exchanges of lipids between the ER and the PM both for the delivery of newly synthesized lipids and for the return to the ER of lipid catabolites for metabolic recycling (Guillén-Samander and De Camilli, 2023).

A protein that functions both as an ER/PM tether and as a lipid transporter is TMEM24 (also called C2CD2L), which is primarily expressed in neurons and pancreatic β-cells (Lees et al., 2017; Sun et al., 2019; Xie et al., 2022). TMEM24 is anchored into the ER membrane via an N-terminal transmembrane region, which is followed on the cytosolic side by an SMP domain, a C2 domain, and an ∼300-amino acid long region which is predicted to be primarily unstructured, but contain stretches of high conservation, including a C-terminal polybasic motif (PBM) (Lees et al., 2017; Sun et al., 2019). TMEM24 dimerizes via the SMP domain, which is the lipid transport module and harbors glycerolipids. It binds the PM through a charge-based association between its PBM and negatively charged lipids present on the inner leaflet of the PM. This interaction is negatively regulated by the Ca^2+^ and PKC-dependent phosphorylation of serine residues interspersed within the PBM. Accordingly, TMEM24 expressed in neurons or other cells is reversibly released from ER/PM junctions and redistributes throughout the ER in response to cytosolic Ca^2+^ elevations (Lees et al., 2017; Sun et al., 2019; Xie et al., 2022). TMEM24 has a paralog, C2CD2, which in contrast to TMEM24 is broadly expressed in all tissues. While having the same domain structure as TMEM24, C2CD2 lacks the serine residues responsible for the phosphoswitch that controls the PM tethering function of TMEM24 and thus is not released from ER/PM junctions in response to Ca^2+^ elevations (Sun et al., 2019).

While studying the properties of TMEM24 in semiconfluent cultured cell lines, we observed a non-homogenous distribution of TMEM24 between portions of the PM adjacent, or non-adjacent, to other cells. Here, we have elucidated the molecular mechanisms responsible for this heterogenous localization of TMEM24 and have discovered an interaction of TMEM24 with band 4.1 proteins, which in turn link TMEM24, and its paralogue C2CD2, to cell adhesion molecules such as SynCAM 1. We speculate that the lipid transport function of TMEM24 may play a role in the support of the signaling reactions that occur at these sites.

## Results

### Preferential accumulation of TMEM24 and C2CD2 at ER/PM junctions localized at sites of cell–cell contact

**Figure 1.**
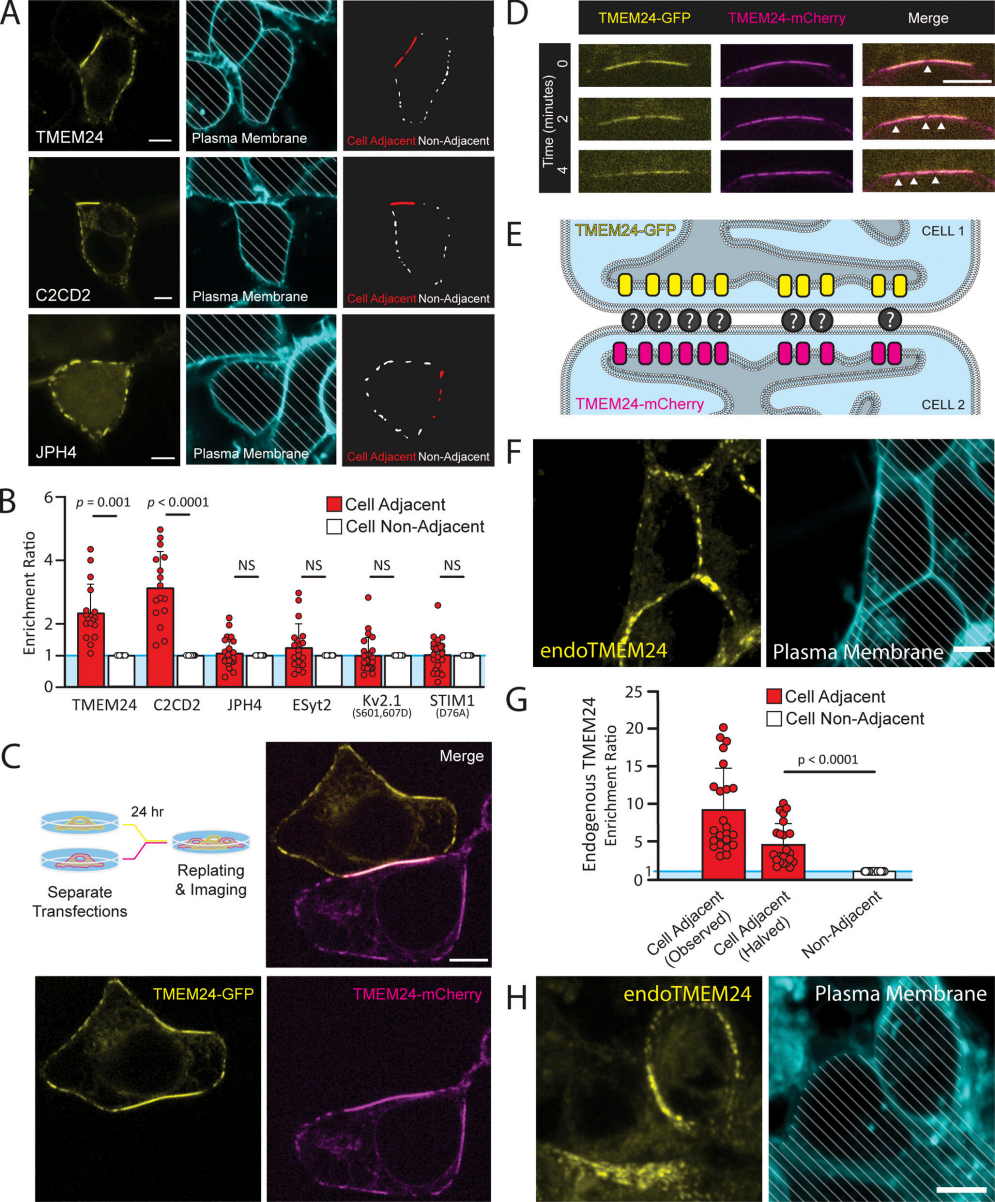
**Large TMEM24-positive ER/PM junctions at cell–cell interfaces. (A)** Left: Spinning disk confocal images of TMEM24-mCherry, C2CD2-eGFP, and JPH4-eGFP in HEK293 cells. Center: plasma membranes labeled with CellBrite 650 dye. Faint diagonal lines have been added to indicate regions of the micrographs occupied by the cells. Right: ER/PM junctions from the left fields with cell-adjacent ER/PM junctions are outlined in red. **(B)** ROIs were drawn around regions of the plasma membrane touching an adjacent cell or facing open space and the mean fluorescence intensity of the indicated proteins within those regions was measured. An enrichment ratio was calculated for each cell by dividing the mean fluorescent intensity at adjacent regions by the mean non-adjacent fluorescent intensities. Student’s *t* test; P values of P = 0.0010 for TMEM24 and P = 7.12902E-07 for C2CD2. **(C and D)** Cells expressing TMEM24-eGFP and TMEM24-mCherry were coplated as illustrated by the diagram. **(C)** TMEM24 expressed in adjacent cells forms symmetrical ER/PM junctions. **(D)** Gaps in the TMEM24 signal (indicated by arrowheads) in one cell are mirrored by gaps in the signal of the adjacent cell. **(E)** Diagram illustrating TMEM24 behavior in adjacent cells expressing TMEM24. **(F)** TMEM24 tagged with GFP at the endogenous locus in IMR32 cells localizes preferentially to the cell–cell interface. **(G)** Quantification of the enrichment ratio for mean fluorescence of TMEM24 tagged at the endogenous locus (endoTMEM24) at regions of IMR32 cells that face adjacent cells or empty space in the dish. As endoTMEM24 signal may stem from both adjacent cells, the observed fluorescence at the adjacent region has been halved and compared against non-adjacent regions (P = 0.000000003, *n* = 24 cells). **(H)** Differentiated IMR32 cells show that endoTMEM24 fluorescence is preferentially localized at sites of cell–cell contact as in the undifferentiated IMR32 cells. In F and H, the plasma membrane was labeled with CellBrite 650 dye. Diagonal lines indicate regions of the micrographs occupied by cells to clearly differentiate these regions from empty spaces. Scale bars = 5 μm.

**Figure S1.**
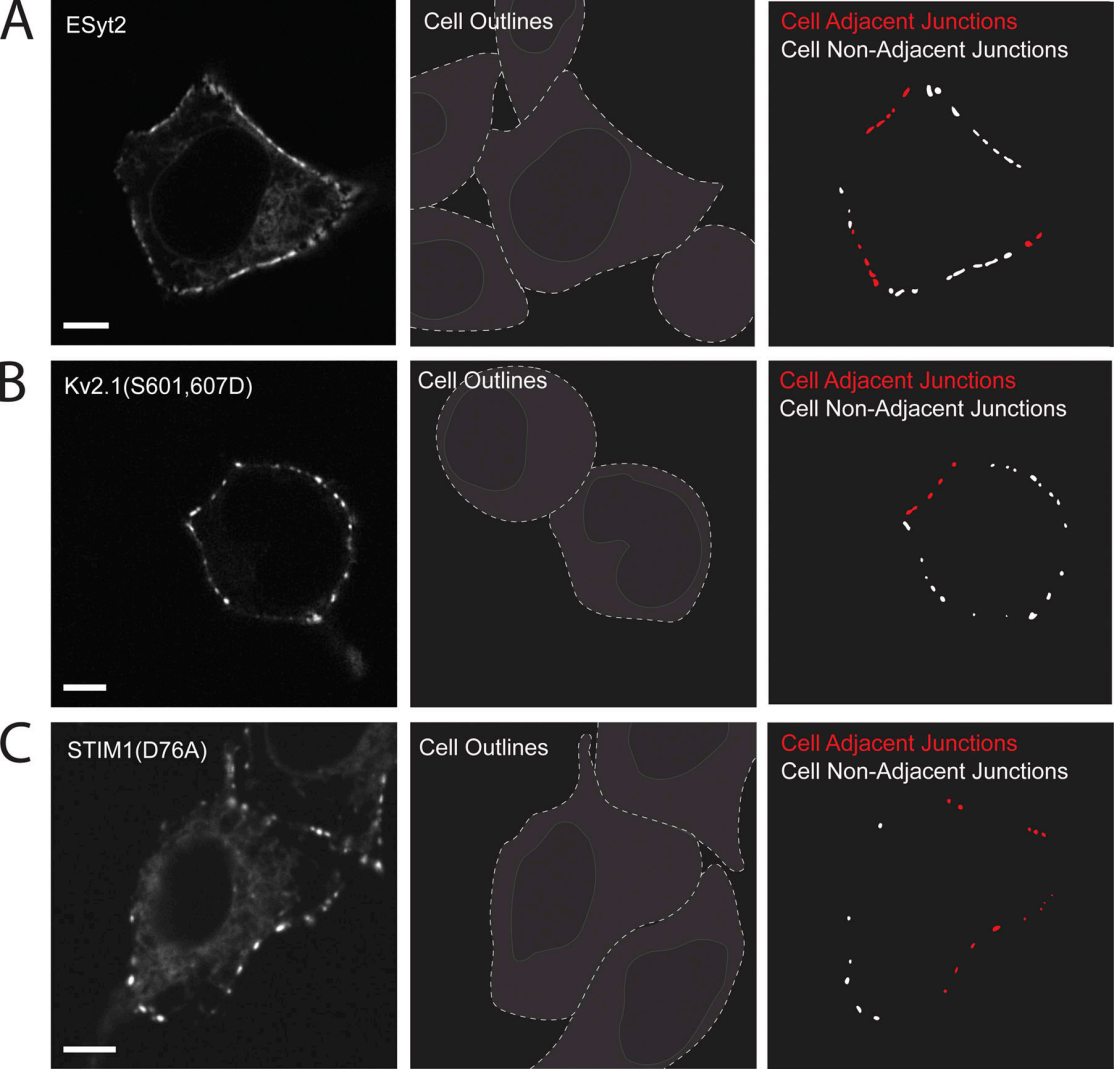
**Several ER/PM tethers tested other than TMEM24 and C2CD2 display no preference for cell-adjacent ER/PM junctions.** ER/PM junctions positive for exogenously expressed E-Syt2-eGFP (A), Kv2.1(S601,607D-eGFP (B), or YFP-STIM1(D76A) (C) in HEK293 cells show no differences in size depending on cell adjacency. Quantification of this data can be found in Fig. 1 B. Plasma membranes were determined using DiI and cell regions traced for ease of identification. Scale bars = 5 μm.

When TMEM24-mCherry or its paralog C2CD2-eGFP were expressed in HEK293 cells, they localized at patches along the PM with the expected pattern of ER/PM junctions. However, such patches were significantly larger and more intense at sites of cell–cell contact (Fig. 1 A). Often these larger junctions spanned the entirety of the cell–cell interface in our overexpression system, which is expected to result in the expansion of endogenous ER/PM contacts. The preferential accumulation to sites where the PMs of two cells are in close apposition was unique to TMEM24 and C2CD2. Other ER/PM tethering proteins that we tested, such as Junctophilin-4 (JPH4-eGFP), extended synaptotagmin-2 (eGFP-E-Syt2) as well as mutant forms of Kv2.1 or STIM1, which constitutively localize at ER/PM contacts [Kv2.1(S601,S607D-eGFP) and YFP-STIM1(D76A)], showed no differences based on the presence of an adjacent cell (Fig. 1 A and Fig. S1). To quantify the enrichment of each protein tested at cell adjacent sites, we calculated an “enrichment ratio.” This ratio was calculated by measuring the mean fluorescence intensity at regions adjacent to another cell and regions not adjacent to another cell and dividing both those values by the mean fluorescence intensity of the non-adjacent region. This results in an enrichment ratio of 1 for all non-adjacent regions and enrichment ratios higher or lower than 1 for adjacent regions based on whether proteins of interest prefer or avoid such regions (see Fig. 1 B; see Materials and methods for additional detail). While other tethers had an enrichment ratio for their adjacent regions that was not significantly different from their cell non-adjacent regions, both TMEM24 and C2CD2 had significantly higher enrichment ratios for their cell-adjacent regions (P = 0.0010 for TMEM24 and P = 7.12902E-07 for C2CD2).

**Figure S2.**
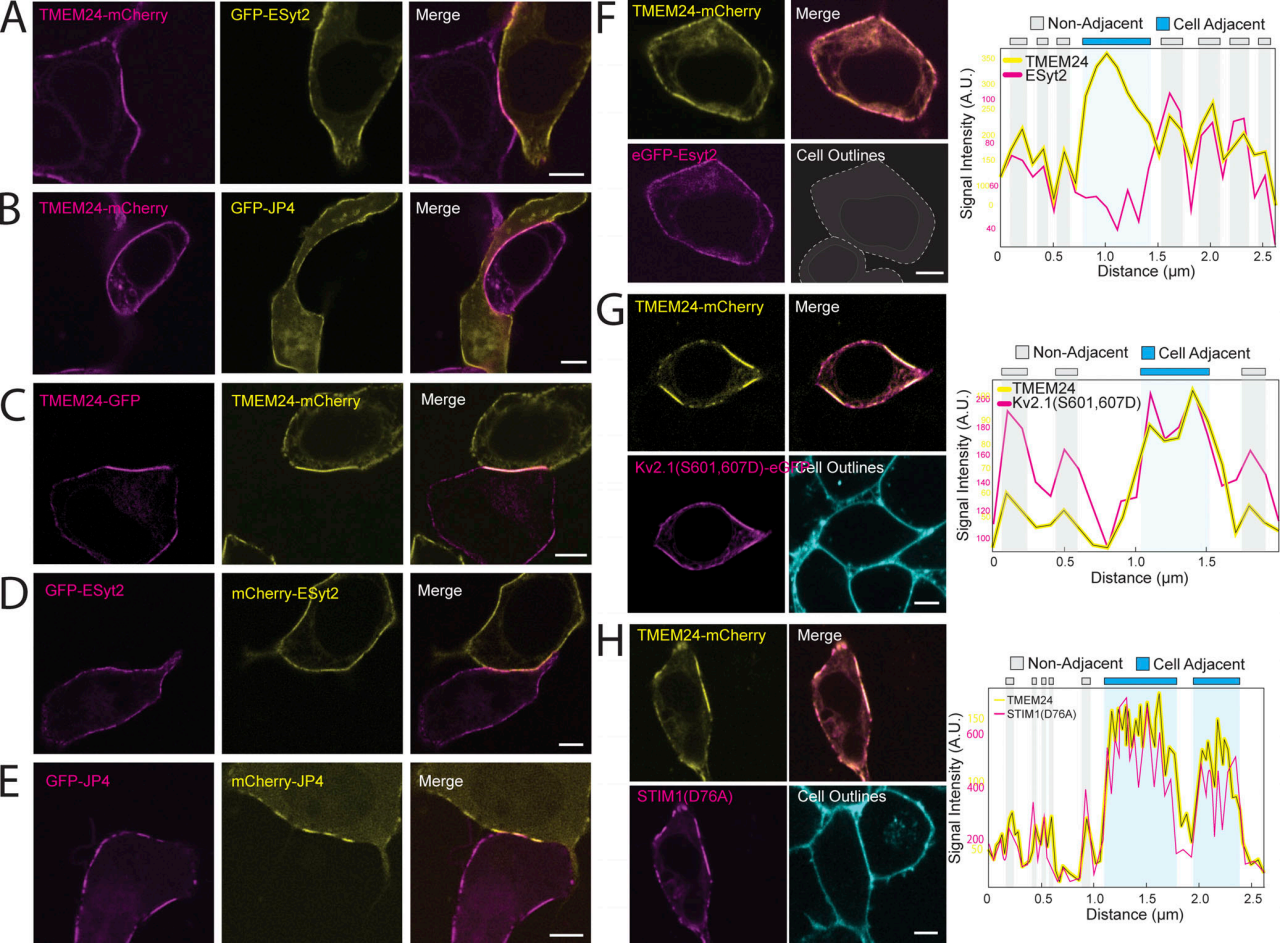
**TMEM24, but not other ER/PM tethers, concentrate as cell–cell junctions.** HEK293 cells. **(A and B)** TMEM24 expressed in one cell generates enlarged ER/PM junctions that are not mirrored by eGFP-ESyt2-positive or eGFP-JPH4-positive junctions in directly adjacent cells. **(C)** The accumulation of TMEM24-mCherry expressed in one cell at a cell-adjacent ER/PM junction is mirrored by the accumulation of TMEM24-GFP expressed in the adjacent cell (see also Fig. 1 C). **(D)** ER/PM junctions induced by mCherry-ESyt2 and GFP-ESyt2-expressed in two adjacent cells respectively, do not mirror one another across the cell–cell interface. **(E)** mCherry-JPH4 and GFP-JPH4 do not robustly mirror one another across the cell–cell interface although junctions could be found that seemed to be symmetrically opposed. **(F)** eGFP-Esyt2 colocalizes with TMEM24 at ER/PM junctions, but is excluded from the cell adjacent junction where TMEM24-GFP is selectively enriched. Line scans of the fluorescence of the two constructs along the plasma membrane are shown at right. **(G)** Kv2.1(S601,607D)-GFP, a Kv2.1 construct that binds constitutively to the ER protien VAP colocalizes with TMEM24-mCherry at all ER/PM junctions. Line scans of the fluorescence of the two constructs along the plasma membrane are shown at right. **(H)** STIM1(D76A)-GFP, a STIM1 construct that constitutively binds the PM, colocalizes with TMEM24-mCherry at all ER/PM junctions. Line scans of the fluorescence of the two constructs along the plasma membrane are shown at right.

We next expressed TMEM24-eGFP in one set of HEK293 cells, TMEM24-mCherry in another set of HEK293 cells, and after 24 h co-plated the two cell populations together (Fig. 1 C). This protocol allowed us to observe the dynamics of TMEM24-positive ER/PM junctions across neighboring cells. Cell pairs that expressed TMEM24 in both cells formed large symmetrical ER/PM junctions across the cell–cell interface with gaps that appeared in the TMEM24 signal over time of one cell being perfectly mirrored by simultaneous gaps in the signal of the neighboring cell (Fig. 1 D). In contrast, the expression of tagged TMEM24 in one cell and either tagged JPH4 or E-Syt2 in an adjacent cell did not result in such large symmetrical junctions (Fig. S2, A and B). Cell pairs expressing other ER/PM tethers (eGFP-E-Syt2 and mCh-E-Syt2, JPH4-eGFP and JPH4-mCh) also did not generate these robustly expanded symmetrical junctions, though the expression of JPH4 in adjacent cells generated perhaps slight symmetry, which we did not further explore (Fig. S2, D and E). These data strongly suggest that TMEM24 may be part of a direct or indirect complex with endogenously expressed cell adhesion molecule(s) (see Fig. 1 E for model).

To ensure that the localization of TMEM24 was not a result of overexpression, we also analyzed the localization of endogenous TMEM24. To this aim, we used previously generated IMR32 human neuroblastoma cells in which endogenous TMEM24 (endoTMEM24-eGFP) had been tagged at the TMEM24 gene locus by gene editing (Sun et al., 2019). In agreement with the HEK293 cell data, we found that also in IMR32 cells TMEM24 accumulated at ER/PM junctions localized at sites of cell appositions (see Fig. 1 F). We quantified this preference by again calculating the enrichment ratio (Fig. 1 G). However, as TMEM24 fluorescence can be from either of the adjacent cells, we halved the observed value from cell adjacent locations to compare to the non-adjacent regions. TMEM24 fluorescence was significantly greater at cell-adjacent regions of the plasma membrane (Fig. 1 G; P = 0.000000003, *n* = 24 cells). We also treated these IMR32 cells with 2.5 µM bromo-deoxyuridine to induce their neuronal differentiation and again observed a clear preference for sites of cell–cell contact (Fig. 1 H). This demonstrates that the localization of TMEM24 at sites of cell adhesion is not an artifact of an overexpression system and that it is also not unique to HEK293 cells.

### TMEM24 cell-adjacent and cell-non-adjacent ER/PM junctions have distinct properties

**Figure 2.**
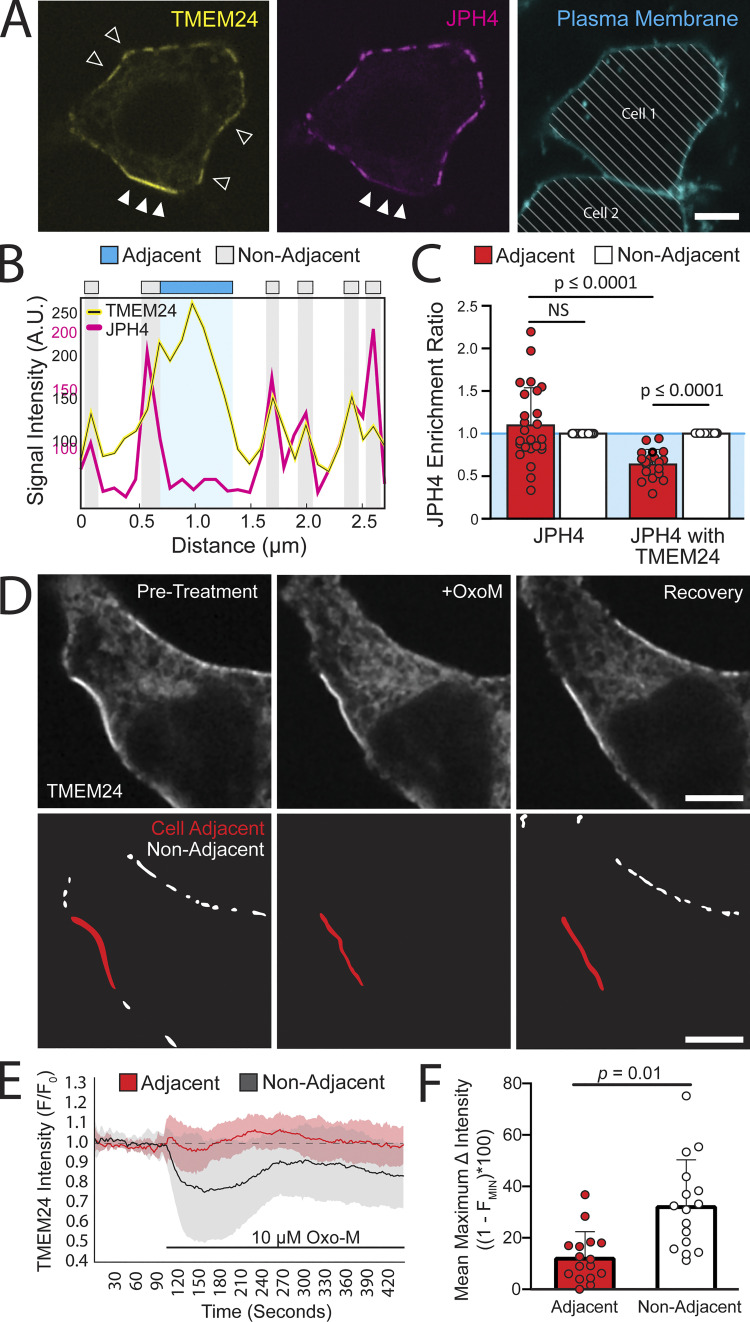
**TMEM24-positive cell-adjacent ER–PM junctions exhibit distinct characteristics. (A)** TMEM24-mCherry and JPH4-eGFP colocalize at ER/PM junctions that are non-cell adjacent (open arrowheads represent a few examples contained within the image) but JPH4 is excluded from TMEM24-induced cell-adjacent ER/PM junctions (solid arrowheads). The plasma membrane was labeled with CellBrite 650 and diagonal lines indicate regions of the micrograph occupied by cells. **(B)** Representative line scan of a cell membrane demonstrating fluorescence increases in both TMEM24 and JPH4 channels at ER/PM junctions with the exception of the cell–cell contact area where TMEM24 signal increases and JPH4 signal is lacking. **(C)** Enrichment ratios of JPH4 expressed alone or with TMEM24. When solely expressed, the adjacent and non-adjacent ratios for JPH4 are not significantly different. Expressed with TMEM24, the adjacent ratio of JPH4 is significantly decreased compared with the non-adjacent value (P = 1.47845E-08) as well as significantly decreased compared with the adjacent value when expressed alone (P = 3.62696E-05). **(D)** Images of TMEM24-mCherry cell adjacent and cell non-adjacent ER/PM junctions before and after addition of 10 μM Oxotremorine-M (OxoM). Junctions that are cell-adjacent and cell non-adjacent are indicated in lower panels. **(E)** TMEM24 response to treatment of 10 μM OxoM separated by cell-adjacent and cell non-adjacent TMEM24 fluorescence over time and normalized to an average pre-treatment fluorescence level. **(F)** Maximal average fluorescence change in TMEM24 signal at cell-adjacent and cell non-adjacent ER/PM junctions. Student’s *t* test returned a value of P = 0.01. All images are from HEK293 cells and were acquired by confocal microscopy. Scale bars = 5 μm.

Co-expressing TMEM24 with other ER/PM tethers revealed molecular heterogeneity between cell-adjacent and cell-non-adjacent ER/PM junctions. JPH4-eGFP and eGFP-E-Syt2 colocalized with TMEM24-mCherry at ER/PM junctions that are cell-non-adjacent but were excluded from TMEM24–mCherry-positive ER/PM junctions localized at sites of cell–cell interfaces (Fig. 2, A and B; and Fig. S2 F). This resulted in an enrichment score for JPH4 that was significantly <1 when coexpressed with TMEM24 as less JPH4 protein is now observed at the adjacent region compared with the non-adjacent region (Fig. 2 C; P = 1.47845E-08). In contrast, Kv2.1(S601, 607D)-GFP and YFP-STIM1(D76A) colocalized with TMEM24-mCh at both cell-adjacent and cell-non-adjacent junctions (Fig. S2, G and H).

We previously showed that the localization of TMEM24 at the PM is regulated by cytosolic calcium. Specifically, we found that calcium/PKC-dependent phosphorylation of basic amino acid residues within the C-terminal polybasic motif of TMEM24 disrupts its binding to the cytosolic surface of the PM (which is enriched in negatively charged phospholipids) and induces its redistribution throughout the ER. Conversely, dephosphorylation of the same residues by the phosphatase calcineurin/PP2B results in the rapid reassociation of TMEM24 with the PM (Lees et al., 2017; Sun et al., 2019). Consistent with these findings, we have now observed that treatment with Oxo-M of HEK293 cells expressing the M1 receptor to induce elevation of cytosolic calcium mediated by PLC activation and IP3 generation resulted in the rapid shedding of TMEM24 from the PM at cell-non-adjacent ER/PM junctions. In contrast, the localization of TMEM24 at junctions that are cell-adjacent was resistant to Oxo-M treatment in these cells (Fig. 2, D–F). This demonstrated that TMEM24 pools at cell-adjacent and cell-non-adjacent junctions responded differently to external stimuli.

### A β-sheet within the TMEM24 C-terminal region is necessary and sufficient for localization at sites of cell–cell contacts

**Figure 3.**
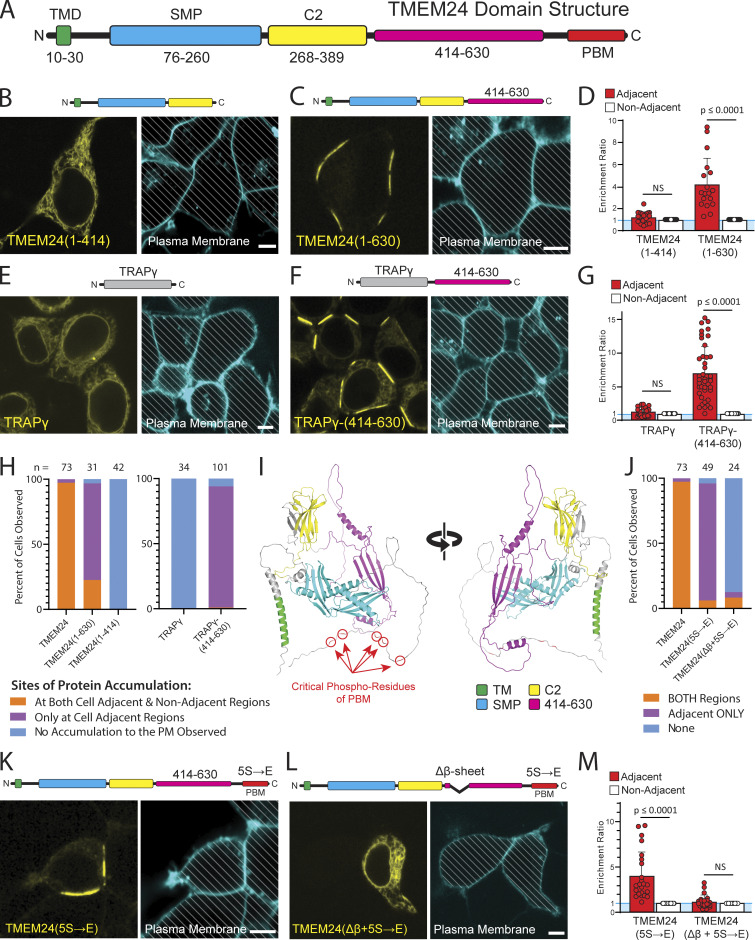
**A β-sheet within the TMEM24 C-terminus is responsible for its targeting to cell-adjacent ER/PM junctions.** HEK293 cells. **(A)** TMEM24 domain architecture. TMD = transmembrane region; PBM = polybasic motif. **(B)** Fragment 1- 414 is diffusely ER localized and does not localize to ER/PM junctions at any location. Representative example of *n* = 42 cells. **(C)** Fragment 1–630 is localized at ER/PM junctions at sites of cell–cell contact. Representative example of *n* = 31 cells. **(D)** Quantification of the enrichment ratios for TMEM24(1–414) and TMEM24(1–630) fragments (P = 0.10388 and P = 2.80624E-05, respectively). **(E)** The TRAPγ-eGFP has a diffuse ER localization. Representative example of *n* = 34 cells. **(F)** TRAPγ-(414–630)-eGFP hybrid construct localizes to ER/PM junctions at sites of cell–cell contact. Representative example of 101 cells. **(G)** Quantification of the enrichment ratios for TRAPγ and TRAPγ-(414–630) (P = 0. 0.1306 and P = 3.03836E-12, respectively). **(H)** Percentages of cells expressing TMEM24, TMEM24(1–414), TMEM24(1–630), TRAPγ, and TRAPγ-(414–630) with accumulation of these proteins at the regions indicated. Percentages are as follows for both regions, adjacent only, and no accumulation, respectively: for TMEM24 97.2%, 2.7%, 0%, *n* = 73; for TMEM24(1–630) 22.6%, 74.2%, 3.2%, *n* = 31; for TMEM24(1–414) 0%, 0%, 100%, *n* = 42; for TRAPγ 0%, 0%, 100%, *n* = 34; for TRAPγ-(414–630) 1.0%, 93.0%, 6.0%, *n* = 101. **(I)** Alphafold predicted structure of a TMEM24 monomer in two different orientations. **(J)** Percentages of cells expressing TMEM24, TMEM24(5S→E), and TMEM24(Δβ + 5S→E) with the accumulation of these proteins at the regions indicated. Percentages are as follows for both regions, adjacent only, and no accumulation, respectively for TMEM24 97.2%, 2.7%, 0%, *n* = 73; for TMEM24(5S→E) 6.1%, 89.8%, 4.1%, *n* = 49; for TMEM24(Δβ + 5S→E) 8.3%, 4.2%, 87.5%, *n* = 24. **(K)** A full-length TMEM24 with the 5S→E mutations in the PBM domain is selectively localized at cell-adjacent regions of the PM. Representative example of *n* = 49 cells. **(L)** Disruption of both the PBM (accomplished via the 5S→E mutation) and the TMEM24 C-terminal β-sheet interferes with TMEM24’s ability to localize at any ER/PM junction. Representative example of *n* = 24 cells. **(M)** Quantification of the enrichment ratios for TMEM24(5S→E) and TMEM24(Δβ + 5S→E) (P = 1.51225E-05 and P = 0.168213084, respectively). The plasma membranes for all micrographs in the figure were labeled with CellBrite 650 dye and diagonal lines indicate regions occupied by cells. Scale bars = 5 μm.

**Figure S3.**
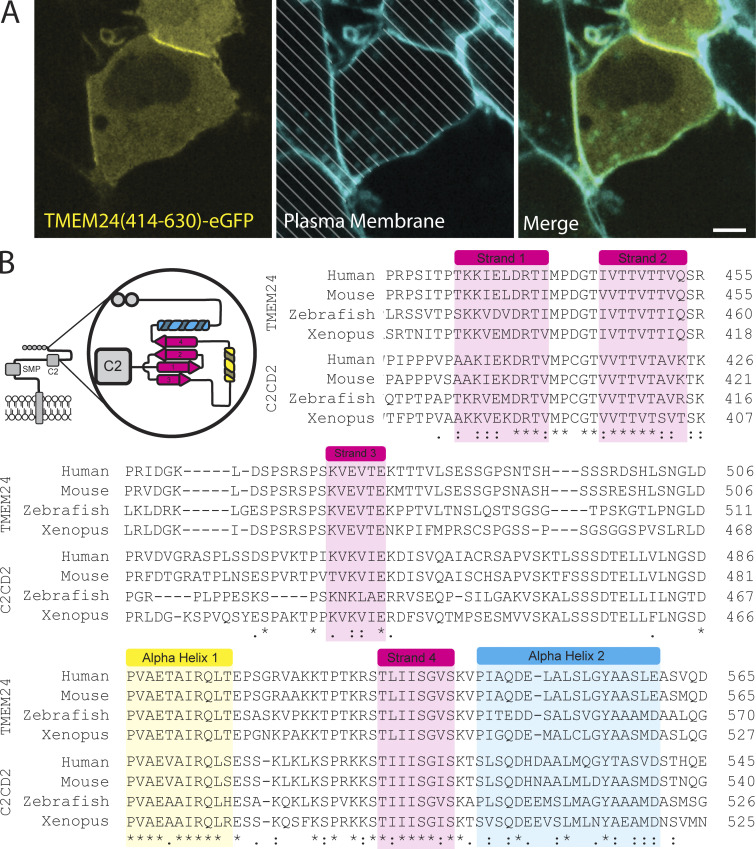
**A C-terminal fragment of TMEM24 that contains a conserved small β-sheet is a soluble protein that accumulates at cell–cell junctions. (A)** The 414–630 C-terminal portion of TMEM24 is a soluble cytosolic protein that concentrates at cell–cell contacts in HEK293 cells. Cell membranes were labeled with CellBrite 650 and diagonal lines indicate regions of the micrographs occupied by cells to clearly differentiate these regions from empty spaces. Scale bar = 5 µM. **(B)** Sequence alignments of portions of the C-terminal regions of TMEM24 and C2CD2 across species demonstrating conservation of predicted α-helixes and β-strands. The cartoon at the top left shows a schematic view of a TMEM24 monomer, with an enlarged view of the Alphafold predicted structural motifs (colored) within its 414–630 amino acid region.

Truncation and internal deletion mutations of TMEM24 were generated to identify the region(s) responsible for its localization at sites of cell–cell contacts (see schematic domain representation of TMEM24 in Fig. 3 A) A construct comprising amino acids 1–414 and including the ER transmembrane sequence and the SMP and C2 domains was localized throughout the ER and failed to accumulate at any ER/PM junction (Fig. 3 B), while a longer construct (a.a. 1–630) accumulated at ER/PM junctions selectively at sites of cell–cell contact (Fig. 3 C and quantified in Fig. 3 D). As the 1–630 construct lacks the phosphoregulated PBM (a.a. 666–706) previously shown to interact with the acidic leaflet of the PM bilayer (Lees et al., 2017; Sun et al., 2019), this finding suggested that TMEM24 contained an additional PM tethering motif within its 414–630 region allowing it to form junctions specifically at cell–cell interfaces. To confirm this hypothesis, we generated a hybrid construct comprising the TMEM24 414–630 region fused to the ER protein TRAPγ-eGFP. TRAPγ is a component of the translocon complex that localizes diffusely and homogenously throughout the ER when expressed alone (Fig. 3 E). The fusion protein, TRAPγ-TMEM24(414–630)-eGFP, not only accumulated at ER/PM junctions but did so specifically at sites of cell–cell contact (Fig. 3 F and quantified in Fig. 3 G). The 414–630 fragment expressed alone also concentrated at cell–cell contacts (Fig. S3 A). As the enrichment ratios of these proteins (Fig. 3, D and G) as defined above do not fully capture differences in their localizations, we also calculated the percentages of cells showing accumulation of proteins at the PM of both cell-adjacent and cell-non-adjacent regions, those with accumulations only at the PM of cell adjacent regions, and those with no observable accumulation at the PM (Fig. 3 H). While TMEM24 is enriched at cell-adjacent regions (demonstrated by the enrichment analysis), it will accumulate at ER/PM junctions at both cell-adjacent and cell-non-adjacent regions (97.3% of 73 transfected cells observed for this experiment had TMEM24 accumulation in both regions). However, the majority of TMEM24(1–630) accumulated only at regions that were adjacent to another cell (74.2% of 31 transfected cells observed). TMEM24(1–414) remained diffusely localized to the ER with no accumulation (100% of 42 transfected cells observed). TRAPγ localized to the ER (100% of 34 transfected cells observed), but the addition of TMEM24 a.a. 414–630 resulted in the accumulation of the hybrid construct only to cell adjacent regions (93% of 101 transfected cells observed). These data support the idea that TMEM24 can form both generally localized ER/PM junctions via the PBM as well as highly targeted cell adjacent junctions that are dependent on its 414–630 region.

AlphaFold predictions suggest that the 414–630 region of TMEM24 is predominantly disordered, but contains three short, structured a.a. sequences: two α-helices and one β-sheet (Fig. 3 I) (Jumper et al., 2021). Disrupting the PBM of TMEM24 with five serine to glutamic acid substitutions (5S→E) abolished the interactions of this region with the acidic cytosolic leaflet of the PM (Sun et al., 2019) and resulted in a protein that localized only to ER/PM contacts at cell–cell interfaces (Fig. 3, J, K, and M). An internal deletion of the first three strands of the β-sheet, in combination with the (5S→E) mutation, completely disrupted TMEM24’s ability to bind the PM, suggesting that the β-sheet is necessary for its localization at sites of cell–cell contact (Fig. 3, J, L, and M). AlphaFold predicts a similar β-sheet in the corresponding region of C2CD2. These regions of both TMEM24 and C2CD2 are highly conserved across higher organisms (Fig. S3 B).

### Identification of TMEM24 interactors via APEX2 proximity biotinylation

To identify potential PM protein interactors for TMEM24 that may be responsible for its targeting to sites of cell adhesion, we turned to APEX2-based proximity biotinylation. In the presence of H_2_O_2_, APEX2-conjugated proteins generate rapidly diffusing biotin radicals that biotinylate electron-rich amino acid residues of nearby proteins. As these radicals have extremely short half-lives (<1 ms), only proteins in the immediate vicinity (∼20 nm) of the APEX2 protein during H_2_O_2_ treatment are biotinylated. Biotinylated proteins can be subsequently purified via streptavidin-based affinity purification and identified by mass spectrometry. This approach has already been successfully used for interrogating the proteome of the ER/PM junction (Jing et al., 2015; Johnson et al., 2018) as well as other cellular subcompartments such as the synaptic cleft (Cijsouw et al., 2018; Loh et al., 2016).

**Figure 4.**
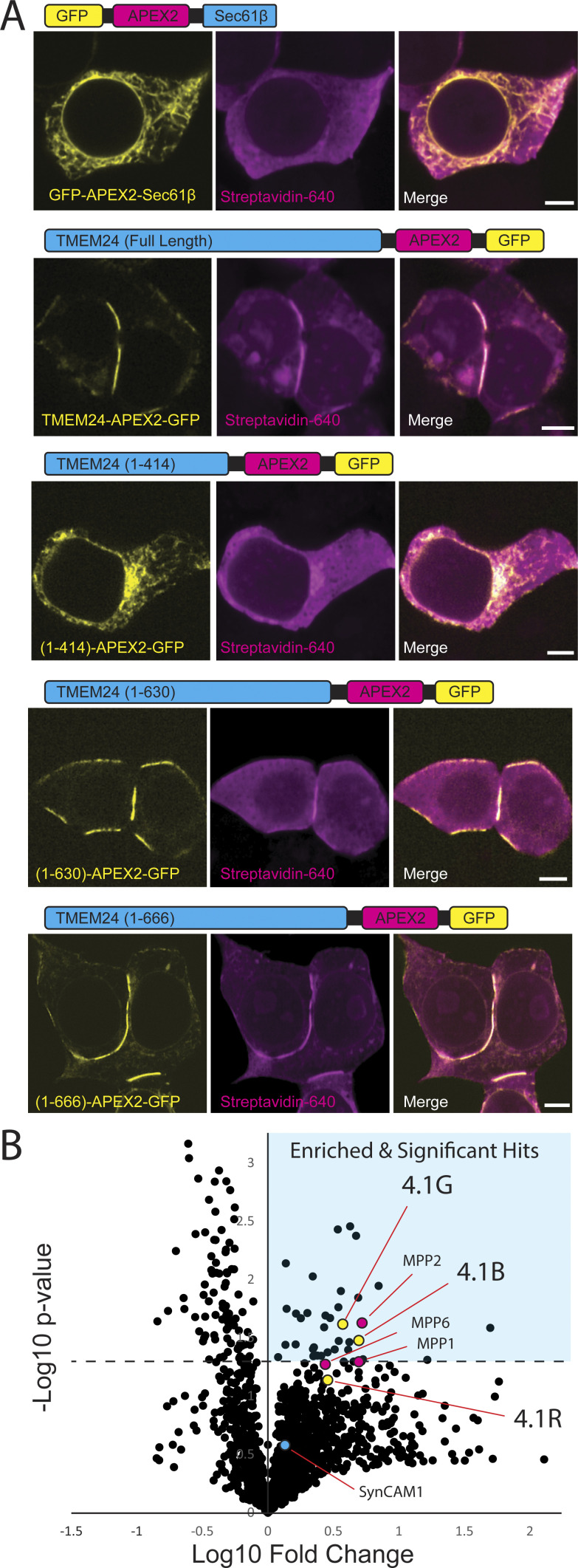
**APEX2 proximity biotinylation identifies TMEM24 protein neighbors. (A)** Schematic representations and confocal images of APEX2 conjugated proteins expressed in HEK293 cells and used in the APEX screen. After the APEX reaction cells were fixed, labeled with CF640R streptavidin to visualize biotinylated proteins and imaged via confocal microscopy. **(B)** Volcano plot of proteins identified via mass spectrometry after streptavidin-based purification. Significantly enriched proteins are found in the upper right quadrant indicated by cyan. Student’s *t* test values for 4.1G, 4.1B, and 4.1R were found to be P = 0.024, P = 0.033, and P = 0.074, respectively. Scale bars = 5 μm.

**Figure S4.**
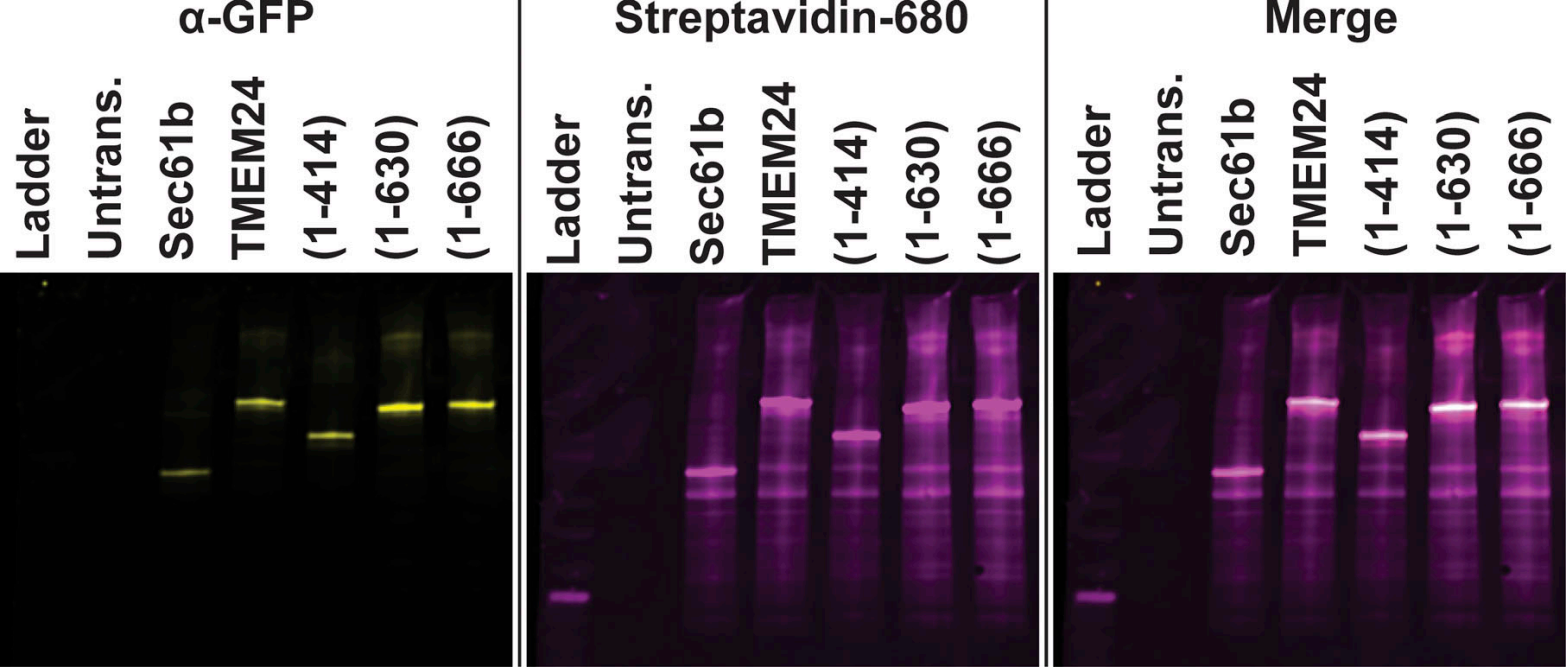
**Streptavidin affinity-purification and localization of the identified band 4.1 proteins.** Anti-GFP western blots and streptavidin overlay of material affinity-purified on streptavidin bead. Numbers in parenthesis indicate amino acid boundaries of TMEM24 fragments used. Note the high degree of self-biotinylation for each construct. Source data are available for this figure: SourceData FS4.

Full-length TMEM24 fused to APEX2 and several other control constructs were generated (Fig. 4 A). All these constructs were additionally tagged with eGFP to validate their correct targeting by fluorescence microscopy (Fig. 4 A). eGFP-APEX2-Sec61β and TMEM24(1–414)-APEX2-eGFP provided controls for diffuse ER localization. TMEM24(1–630)-APEX2-eGFP and TMEM24(1–666)-APEX2-eGFP, which both preferentially localized at cell-adjacent ER/PM junctions, also served as valuable internal controls that should biotinylate an identical or near-identical set of proteins. Untransfected HEK293 cells were used to discriminate between the signal produced by the transfected constructs and endogenous biotinylation. After the APEX2 reaction, a pool of cells was fixed and labeled with a streptavidin-conjugated far-red probe. Microscopy analysis of these cells confirmed that cell regions intensely positive for the biotinylation signal were consistent with the subcellular localization of each eGFP-labeled construct (Fig. 4 A). Another pool of cells was homogenized, biotinylated proteins were affinity-purified on streptavidin beads, and the affinity-purified material was analyzed by SDS-PAGE. Streptavidin and anti-GFP immunolabeling of this material revealed not only strong self-biotinylation of the APEX2-constructs as expected (identified by anti-GFP western blotting) but also a wide range of additional biotinylated protein bands (Fig. S4). To detect the nature of these other proteins, affinity-purified samples were submitted for mass spectrometry analysis in triplicate (three independent biological replicates).

The lists of proteins identified by mass spectrometry in the different sets of affinity-purified biotinylated material were compared to identify hits specifically enriched in the samples biotinylated by full-length TMEM24-APEX2-eGFP, TMEM24(1–630)-APEX2-eGFP, and TMEM24(1–666)-APEX2-eGFP constructs, relative to controls lacking the a.a. 414–630 region (see Fig. 4 B for volcano plot of grouped data and Table S1 for full list of identified proteins). Among the 36 significant hits (see Materials and methods), there were two of the four band 4.1 proteins (4.1G and 4.1B) encoded by the human genome, which ranked at position 18 and 21 (P = 0.024, P = 0.033), respectively. A third band 4.1 protein, 4.1R, was also among the proteins enriched in constructs containing the a.a. 414–630 region (it ranked at position 64) although its enrichment did not reach significance (P = 0.074). The fourth band 4.1 family member, 4.1N, was not identified in any of our experimental or control APEX conditions, and we did not test for endogenous expression in our HEK293 cells. Interestingly, TMEM24 was previously found as a potential interactor of 4.1R using a rat kidney yeast two-hybrid screen (Calinisan et al., 2005), although this interaction was not further validated. We note that the list of 36 significant hits includes desmoglien-2 (DSG-2), a protein that was recently shown to be part of symmetrical desmosome-endoplasmic reticulum subcellular complexes at cell–cell contacts (Bharathan et al., 2023).

### Band 4.1 proteins interact with the β-sheet of TMEM24 via their C-terminal domain

**Figure 5.**
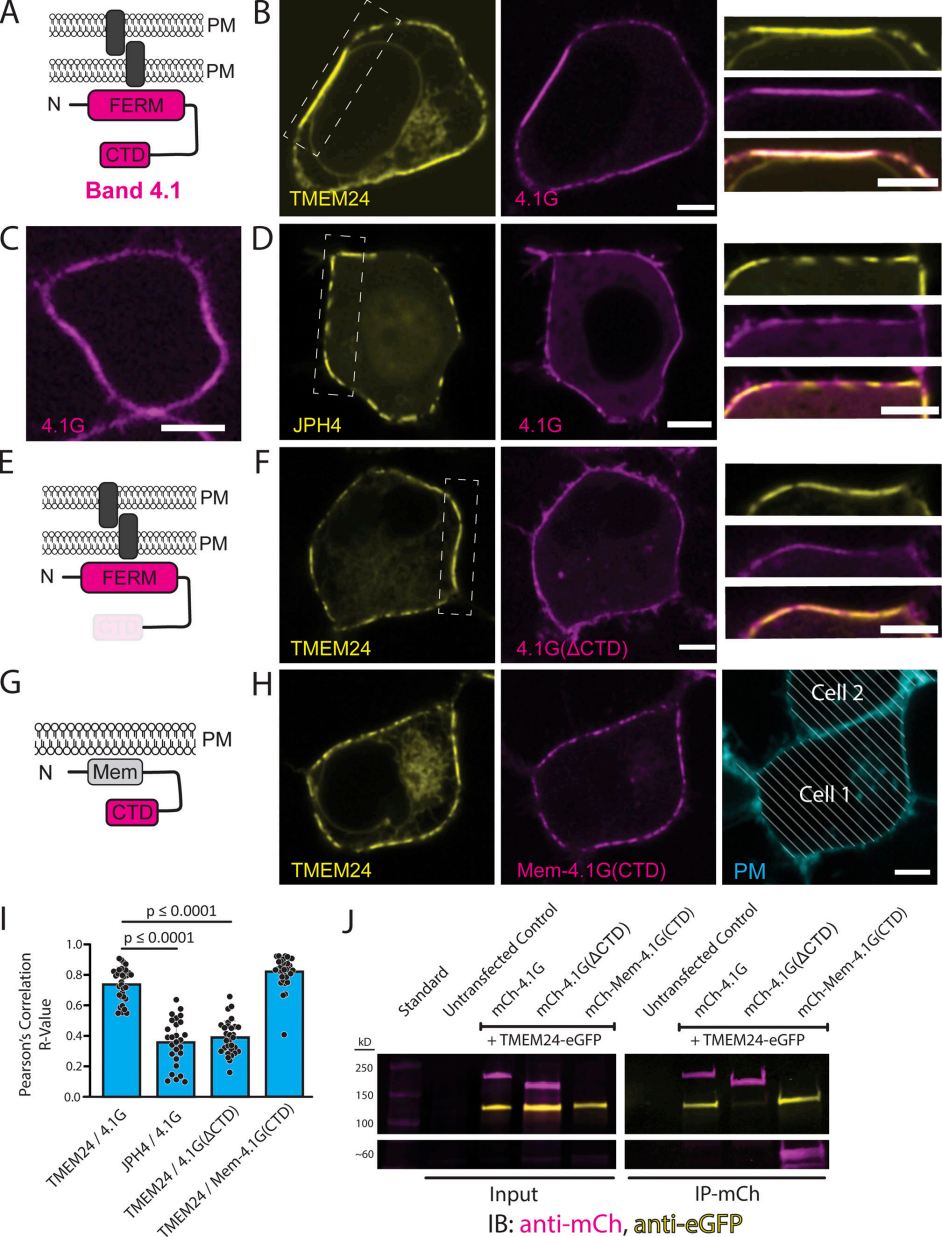
**Band 4.1 proteins bind TMEM24 via an interaction between a TMEM24 β-sheet motif and the C-terminal domain of Band 4.1. (A)** Diagram of Band 4.1 family proteins (a cell adhesion protein is indicated by a gray rectangle). **(B)** Confocal images of HEK293 cells expressing TMEM24-eGFP and mCherry-4.1G. High magnification of the boxed region are shown at right. Representative example of *n* = 34 cells. **(C)** mCherry-4.1G expressed in a HEK293 cell accumulates at the plasma membrane with enrichment at regions adjacent to a neighboring cell. **(D)** Co-expression of JPH4-eGFP and mCherry-4.1G in HEK293 cells result in no co-enrichment at the ER/PM contact. Representative example of *n* = 27 cells. **(E)** Diagram of 4.1G(ΔCTD) with cell adhesion molecule as gray rectangle. **(F)** Confocal images of HEK293 cells expressing TMEM24-eGFP and mCherry-4.1G(ΔCTD). High magnification of the boxed region are shown at right. Representative example of *n* = 31 cells. **(G)** Diagram of the Mem-mCherry-4.1G(CTD) construct. **(H)** Confocal images of the corresponding construct co-expressed with TMEM24 in HEK293 cells. The two proteins are colocalized at ER/PM junctions, but the strong accumulation of TMEM24 at sites of cell–cell contacts is no longer observed. The panel at right shows the location of a neighboring cells stained with CellBrite 650. Representative example of *n* = 33 cells. **(I)** Pearson’s correlation analysis of the colocalization of the indicated constructs. TMEM24 and 4.1G are significantly more colocalized (R_obs_ = 0.74) than JPH4 and 4.1G (R_obs_ = 0.36; P = 1.96443E-14) and TMEM24 and 4.1G(ΔCTD) (R_obs_ = 0.39; P = 1.75766E-19). TMEM24 and Mem-4.1G(CTD) also display high colocalization with one another (R_obs_ = 0.82). **(J)** Anti-mCherry immunopurification from extracts expressing TMEM24-eGFP with mCherry-4.1G, mCherry-4.1G(ΔCTD) or mCherry-Mem-4.1G(CTD) showing co-enrichment of TMEM24-eGFP with mCherry-4.1G and mCherry-Mem-4.1(CTD) but not with mCherry-4.1(ΔCTD). Source data are available for this figure: SourceData F5.

Band 4.1 family proteins, which lack a transmembrane region, are key components of the PM-associated cytoskeleton in all cells (Baines et al., 2014). They have a similar domain architecture (see Fig. 5 A) with the highest degree of conservation occurring within their N-terminal FERM domains (Four point 1, Ezrin, Radixin, and Moesin) and C-terminal domains (CTD) (Baines et al., 2014). The FERM domain mediates binding to the PM by interacting with a variety of PM-localized proteins and lipids, including cell adhesion proteins such as SynCAM 1/CADM1, CADM4, CD44, and members of the β integrin family (Baines et al., 2014). The enrichment of three band 4.1 family members in the proximity proteome of TMEM24 constructs that accumulate at cell adhesion sites, along with their reported adaptor function for proteins implicated in cell adhesion, prompted us to explore a potential direct interaction of TMEM24 with members of the protein band 4.1 family.

**Figure S5.**
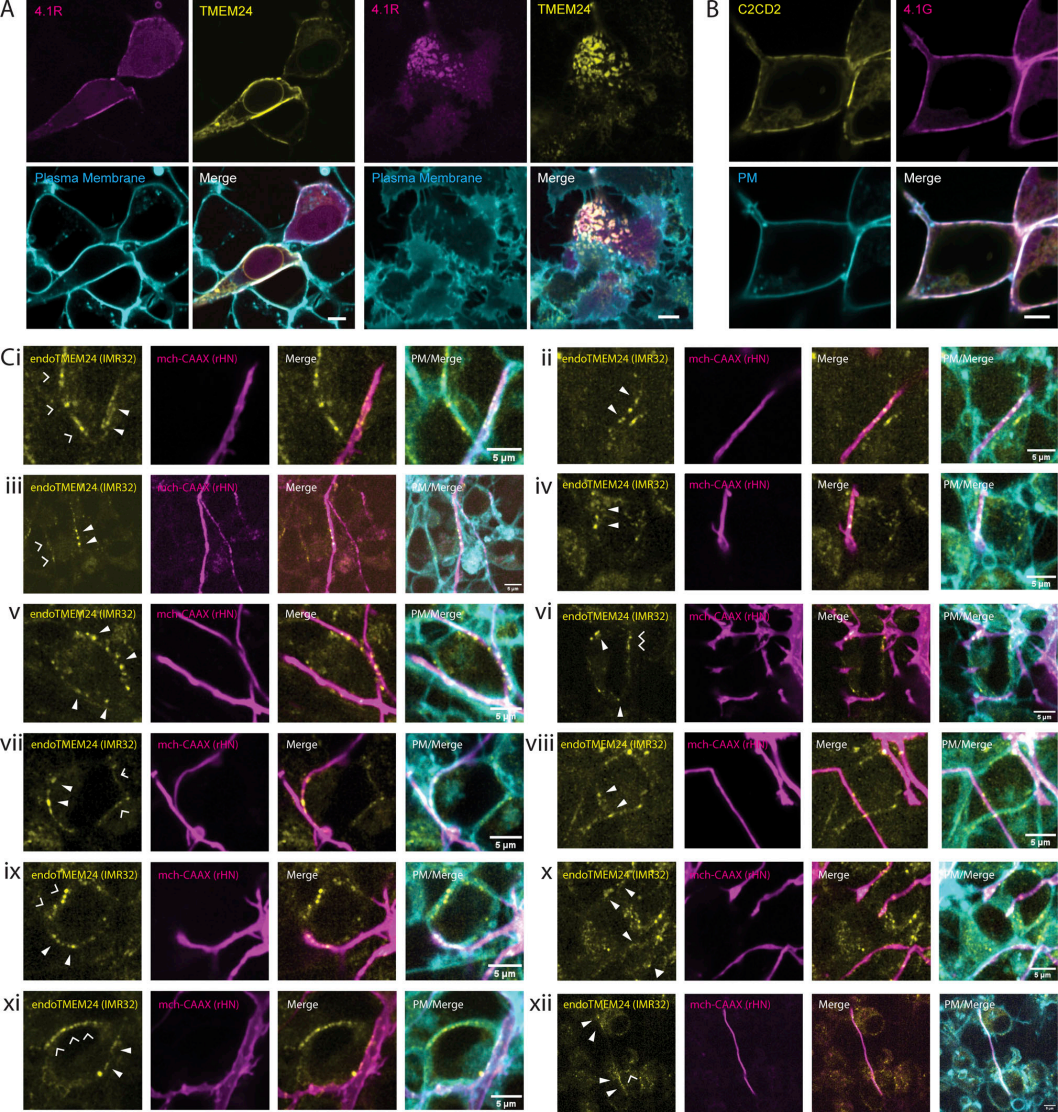
**Colocalization of paralogs and additional images of TMEM24 localization with coplated rat hippocampal neurons. (A)** Colocalization of TMEM24-mCherry with band eGFP-4.1R at ER/PM junctions of HEK293 cells as seen in both a mid-cell confocal z-slice and at the basal surface. **(B)** Colocalization of C2CD2-eGFP with mCherry-4.1G. **(C)** An array of images of IMR32 cells with TMEM24 tagged at the endogenous locus with eGFP that have been coplated with DIV12-14 rat hippocampal neurons transfected with mCh-CAAX. Arrowheads indicate TMEM24-positive ER/PM junctions that are aligned with an mCh-CAAX-positive neuronal process. Chevrons indicated TMEM24-positive ER/PM junctions occurring between adjacent IMR32 cells. Scale bars = 5 µM.

To this aim, we co-expressed either mCherry-4.1G or mCherry-4.1R with TMEM24-eGFP in HEK293 cells (see Fig. 5 B and Fig. S5 A) and found that both proteins precisely colocalized at TMEM24 positive ER/PM contacts. The same results were obtained by co-expressing mCherry-4.1G and C2CD2-eGFP (Fig. S5 B). When expressed alone, protein 4.1G exhibits a diffuse localization across the PM with enrichment at regions where cells are in contact with each other (Fig. 5 C). Co-expressing JPH4 with protein 4.1G resulted in no co-enrichment of 4.1G at JPH4-induced ER/PM contacts (Fig. 5 D), revealing that such co-enrichment is TMEM24 specific and 4.1G does not enrich in general at ER/PM contacts. Additionally, a 4.1G construct that lacks its CTD (mCherry-4.1GΔCTD) did not colocalize with TMEM24 (Fig. 5, E and F), while artificially targeting the CTD of 4.1G to the PM using a PM targeting peptide followed by a short linker [Mem-mCherry-4.1(CTD)] was sufficient for colocalization with TMEM24 (Fig. 5, G and H). With this construct, however, which lacks the 4.1 FERM domain, TMEM24 no longer became concentrated at sites of cell adhesion, suggesting that overexpression of the artificial PM-targeted CTD was outcompeting endogenous band 4.1 proteins for TMEM24 binding. We quantified the degree of colocalization between all the above conditions using a Pearson’s correlation test (Fig. 5 I), which showed significant increases in correlation between TMEM24 and either 4.1G or Mem-4.1G(CTD) compared with the correlation between JPH4 and 4.1G or between TMEM24 and 4.1G(ΔCTD). This demonstrates that the CTD of the band 4.1 proteins is both necessary and sufficient for colocalization with TMEM24. Furthermore, when extracts of HEK293 cells coexpressing TMEM24-eGFP with mCherry-4.1G, mCherry-4.1G(ΔCTD), or mCherry-Mem-4.1(CTD) were incubated with anti-mCherry magnetic beads, eGFP-TMEM24 was affinity-purified alongside mCherry-4.1G and mCherry-Mem-4.1(CTD) but not mCherry-4.1G(ΔCTD) (Fig. 5 J), demonstrating the necessity and sufficiency of the CTD for binding TMEM24.

**Figure 6.**
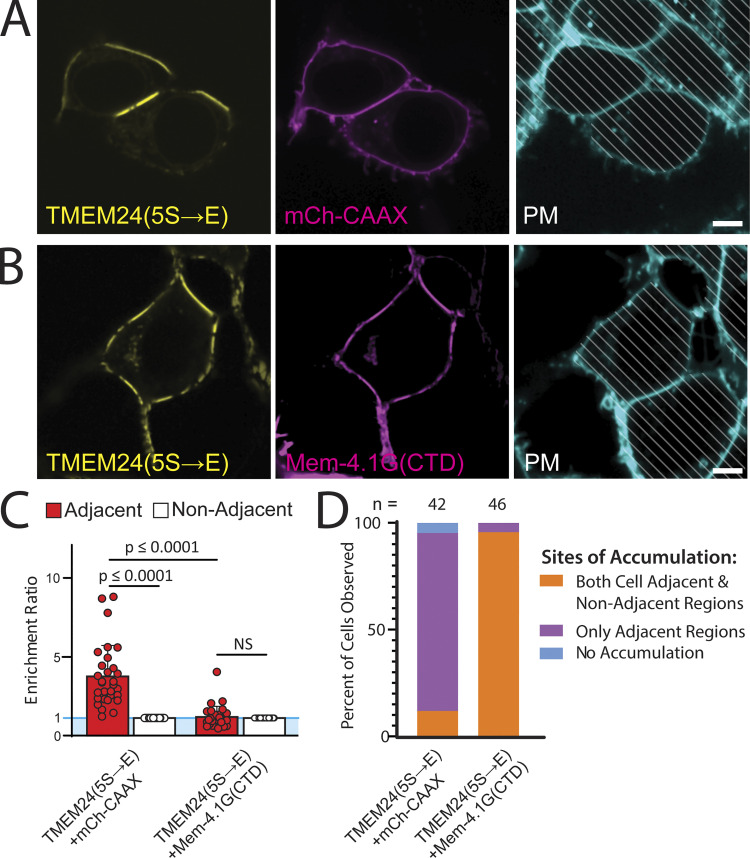
**Overexpression of the membrane bound 4.1G C-terminal domain relocalizes TMEM24 away from cell-adjacent regions. (A)** TMEM24(5S→E)-eGFP expressed with mCherry-CAAX control localizes to regions of the plasma membrane adjacent to a neighboring cell. **(B)** TMEM24(5S→E)-eGFP expressed with mCherry-Mem-4.1G(CTD) localizes to ER/PM junctions diffusely scattered across the entire PM. **(C)** Quantification of the enrichment ratio of TMEM24(5S→E)-eGFP when expressed with either mCherry-CAAX or mCherry-Mem-4.1G(CTD). TMEM24(5S→E)-eGFP expressed with mCh-CAAX is significantly enriched at cell-adjacent regions of the PM (P = 2.43256E-08). TMEM24(5S→E)-eGFP expressed with mCherry-Mem-4.1G(CTD) is not significantly enriched at cell adjacent regions over non-adjacent regions (P = 0.1932). The enrichment ratio for TMEM24(5S→E)-eGFP expressed with mCherry-CAAX at adjacent regions is also significantly higher than when expressed with mCherry-Mem-4.1G(CTD) (P = 7.55685E-08). **(D)** Percentages of cells expressing TMEM24(5S→E)-eGFP with either mCherry-CAAX or mCherry-Mem-4.1G(CTD) with accumulation of these proteins at the regions indicated. Percentages are as follows for both regions, adjacent only, and no accumulation, respectively: for TMEM24(5S→E) + mCh-CAAX 11.9%, 83.3%, 4.8%, *n* = 42; for TMEM24(5S→E) + mCherry-Mem-4.1G(CTD) 95.7%, 4.3%, 0%, *n* = 46.

To further test the possibility that overexpression of the artificially PM-targeted CTD could outcompete endogenous band 4.1 proteins and relocate TMEM24 away from sites of cell adhesion, we turned to the TMEM24(5S→E) construct, which only resides at cell–cell contacts (Fig. 6 A) and co-expressed it with either mCherry-CAAX (as a control) or Mem-mCherry-4.1(CTD). Co-expressing the Mem-4.1G(CTD) construct, but not mCherry-CAAX, drastically altered the localization of TMEM24(5S→E), resulting in its accumulation at ER/PM contacts evenly distributed across the entire PM (Fig. 6, A and B, quantified in Fig. 6, C and D). Collectively, these results strongly support the idea that band 4.1 proteins function as adaptors between TMEM24 (via interactions between the TMEM24 β-sheet motif and the 4.1 CTD) and cell adhesion molecules (via band 4.1 FERM domain-dependent interactions).

### Point mutations within the predicted TMEM24 4.1 binding motif disrupt localization at cell–cell contacts

**Figure 7.**
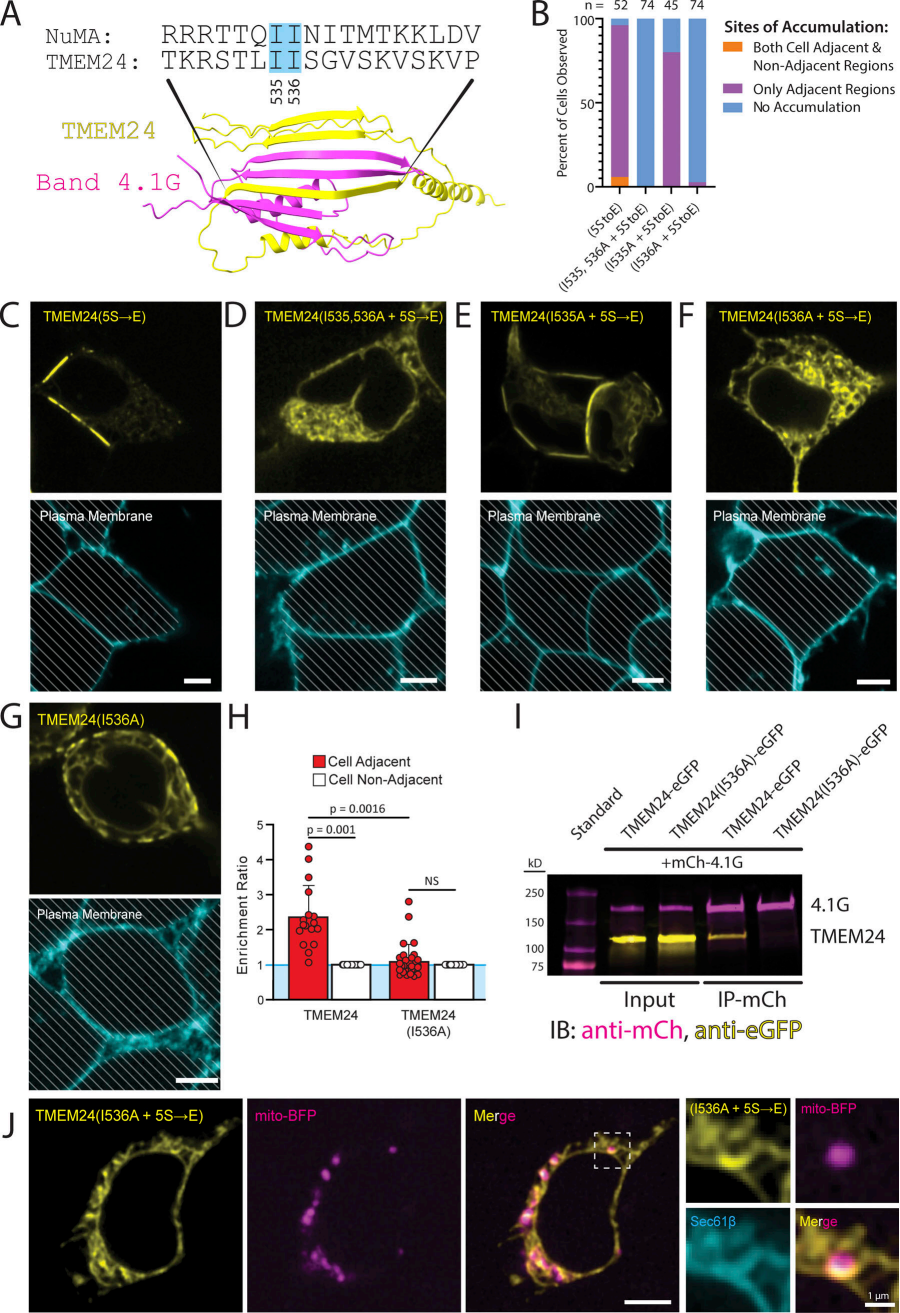
**Point mutations within the 4.1 binding motif of TMEM24 disrupt its targeting to cell–cell junctions. (A)** Alphafold predicted binding between the β-sheet regions of TMEM24 and 4.1G CTD, showing interlocking of their β-strands. The isoleucine residues conserved between TMEM24 and NuMA and mutated to alanine in other panels of the figure are indicated by solid cyan boxes. **(B)** Percent of cells with observed ER/PM contacts upon expression of the constructs indicated. Percentages are as follows for both regions, adjacent only, and no accumulation, respectively: for TMEM24(5S→E) 5.8%, 90.4%, 3.8%, *n* = 52; for TMEM24(I535, 536A + 5S→E) 0%, 0%, 100%; for TMEM24(I535A + 5S→E) 0%, 80%, 20%, *n* = 45; for TMEM24(I536A + 5S→E) 0%, 2.7%, 98.6%, *n* = 74. **(C–F)** Top: representative confocal images of cells expressing the indicated constructs. Below: plasma membranes of the cells shown above were labeled with CellBrite 650. **(G)** Representative confocal image of a cell expressing TMEM24(I536A) (without the additional 5S→E mutation) and of its plasma membrane labeled with CellBrite 650. **(H)** Quantification of the enrichment ratios of TMEM24 and TMEM24(I536A) (as in G) (P = 0.001 and P = 0.1913, respectively, with a significant difference between the ratios of P = 0.0016). **(I)** Anti-mCherry immunopurification from extracts expressing mCherry-4.1G with TMEM24-eGFP or TMEM24(I536A)-eGFP showing co-enrichment of TMEM24-eGFP with mCherry-4.1G, but no co-enrichment of TMEM24(I536A)-eGFP with mCherry-4.1G. **(J)** Representative confocal images of a cell expressing TMEM24(I536A + 5S→E)-eGFP, mCherry-sec61β, and mito-BFP. High magnifications of the boxed region, including the mCherry-sec61β signal (cyan) are shown at right. All cells of the figure are HEK293. Diagonal lines indicate regions of the micrographs occupied by cells. All scale bars = 5 μm unless otherwise noted. Source data are available for this figure: SourceData F7.

As our studies described above had implicated the β-sheet motif within the C-terminal region of TMEM24 in its targeting to sites of cell–cell contact, we used Alphafold multimer (Evans et al., 2021, *Preprint*), an algorithm designed to interrogate protein–protein interactions, to explore the presence in band 4.1 proteins of potential binding interfaces for this motif. This algorithm predicts with strong confidence (ranking confidence score of 0.75) the binding of the β-sheet of TMEM24 to the CTD of 4.1G, which also folds into a small β-sheet, with an intertwining of the β-strands of the two proteins to generate a single chimeric β-sheet (Fig. 7 A). This organization mirrors the one observed between protein 4.1G and the small β-sheet of another known interactor of this protein, NuMA (Hu et al., 2023). In fact, the central β-strands of the β-sheet of both TMEM24 and NuMA share sequence similarity, and both contain a pair of sequential isoleucine residues that are known to be critical for the 4.1G-NuMA interaction (Hu et al., 2023). To test the importance of these isoleucine residues (I535 and I536) in TMEM24, we mutated them to alanine separately or together in the TMEM24(5S→E)-eGFP construct, whose selective localization at cell contacts is dependent on 4.1 proteins. We found that the mutation of isoleucine 535 had no effect on the localization of TMEM24(5S→E), while mutation of isoleucine 536 either alone or in combination with isoleucine 535 was sufficient to abolish the localization of TMEM24 at cell–cell contacts resulting in its diffuse intracellular distribution (Fig. 7, B–F). When the I536A mutation was introduced in WT TMEM24 (without the 5S→E mutations), this protein localized at the smaller ER/PM junctions spread across the entire PM (i.e., those mediated by the PBM) but did not accumulate at cell–cell junctions (Fig. 7, G and H). When extracts of HEK293 cells coexpressing TMEM24-eGFP or TMEM24(I536A)-eGFP with mCherry-4.1G were incubated with anti-mCherry magnetic beads, eGFP-TMEM24 was affinity-purified alongside mCherry-4.1G but not TMEM24(I536A)-eGFP (Fig. 7 I).

Further inspection of cells expressing TMEM24(5S→E) with the additional I536A mutation, i.e., the construct no longer localized at any ER/PM contacts, revealed focal accumulations of this ER protein in the proximity of mitochondria, which had been visualized by co-expression of mito-BFP (Fig. 7 J). Interestingly, TMEM24 was previously reported to concentrate at contacts between the ER and mitochondria upon calcium-induced dissociation from the PM (Xie et al., 2022). Together, our findings and this previous study suggested the existence in TMEM24 of a binding site for mitochondria, which does not involve either the PBM or the 4.1-binding motif, which we did not investigate further.

### TMEM24 can form a complex with band 4.1 proteins and SynCAM 1

We next tested directly the possibility that TMEM24 and band 4.1 proteins could form a complex comprising also a cell adhesion protein. One cell–cell adhesion protein that binds the FERM domain of band 4.1 proteins is SynCAM 1 (also known as CADM1, Necl-2, TSLC-1, and IgSF4) (Baines et al., 2014; Yageta et al., 2002). We chose this protein to assess this possibility as endogenous SynCAM 1 was also a hit in our APEX screen in HEK293 cells, although its average enrichment relative to controls did not reach statistical significance. Moreover, SynCAM 1 in HEK293 cells was reported to form a tripartite complex comprising both band 4.1 proteins and members of the “membrane palmitoylated protein” (MPP) family (MPP1, MPP2, and MPP3) (Sakurai-Yageta et al., 2009) at cell–cell interfaces. Interestingly, three members of this family of MAGUK domain-containing adaptors (MPP1, MPP2, and MPP6) were also hits in our screen for TMEM24 neighbors at sites of cell adhesion (see Fig. 4 B and Table S1), with MPP1 being a statistically significant hit (ranked at position 16; P = 0.024). MPP2 and MPP6 were ranked at positions 43 and 37, respectively, with P values of P = 0.053 and P = 0.0502, respectively.

**Figure 8.**
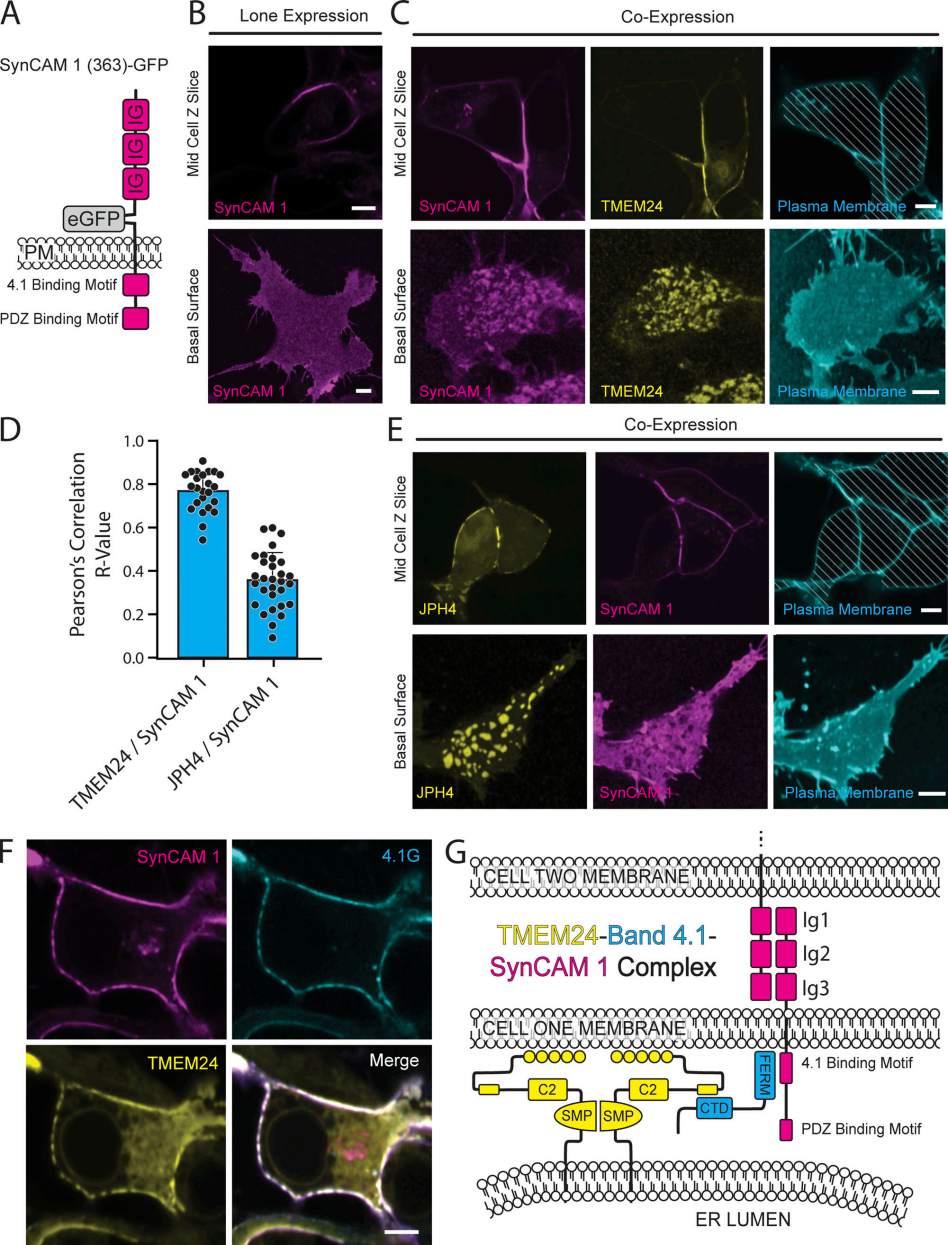
**A TMEM24-4.1-SynCAM1 complex at sites of cell–cell contact. (A)** Schematic of the SynCAM 1(363)-GFP construct. **(B)** Confocal images of SynCAM1(363)-eGFP expressed in HEK293 cells. The upper panel is a slice at mid z level while the lower panel is shown as a basal surface. Representative example of *n* = 22 cells. **(C)** Coexpression of TMEM24-mCherry and SynCAM1(363)-eGFP in HEK293 cells. Images are of a mid-level z-slice (above) or a basal surface (below) of the cell. Plasma membrane labeled with CellBrite 650. Representative example of *n* = 35 cells. **(D)**. Coexpression of JPH4-mCherry and SynCAM1(363)-eGFP in HEK293 cells. The plasma membrane was labeled with CellBrite 650. Representative example of *n* = 28 cells **(E)** Pearson’s correlation R values of comparisons of the SynCAM1(363)-eGFP fluorescence with the fluorescence of either TMEM24-mCherry or mCherry-JPH4 (P = 6.76E-11, TMEM24 *n* = 24, JPH4 *n* = 30). **(F)** Expression of SynCAM1(363)-eGFP, mCherry-4.1G, and TMEM24-Halo labeled with JF646 HaloTag ligand in HEK293 cells. All three proteins colocalize at ER/PM junctions. Representative example of *n* = 74 cells. **(G)** Model of the TMEM24-4.1-SynCAM 1 complex. Diagonal lines indicate regions of the micrographs occupied by cells. Scale bars = 5 μm.

To explore the ability of SynCAM 1 to form a complex comprising TMEM24 at sites of cell adhesion, we expressed SynCAM 1 with an extracellular eGFP tag at amino acid 363 (SynCAM 1(363)-eGFP) in HEK293 cells (see Fig. 8 A for diagram). This internal tag, placed on the extracellular portion of the protein but before the IG domains, leaves the cytosolic sequence of SynCAM 1 unchanged and also does not interfere with the cell adhesion properties of the molecule (Fogel et al., 2007). When expressed alone, SynCAM 1(363)-eGFP was strongly enriched at cell–cell interfaces, although it was also present throughout the PM (note for example its uniform distribution on the basal surface of the cell), possibly due to overexpression (Fig. 8 B). However, when co-expressed with TMEM24-mCh, it co-clustered with this protein (most likely via the adapter role of endogenous band 4.1 proteins) throughout the PM, both at the cell–cell interface as well as at all other ER/PM junctions including those formed at the basal surface (Fig. 8 C). In contrast, control co-expression of JPH4-mCh and SynCAM 1(363)-eGFP led to the exclusion of SynCAM 1 from JPH4-positive ER/PM junctions (Fig. 8, D and E), consistent with the exclusion of 4.1G from such junctions (Fig. 5 D) and the predominant exclusion of JPH4 from cell-adjacent junctions (Fig. 2, A–C). Finally, when expressed together, TMEM24, 4.1G, and SynCAM 1 colocalized as would be expected if they formed a complex (Fig. 8, F and G). These findings support the hypothesis that band 4.1 can function as an adaptor enriching TMEM24 at sites of cell adhesion.

### TMEM24 ER/PM junctions can be found at neuronal cell contact sites

**Figure 9.**
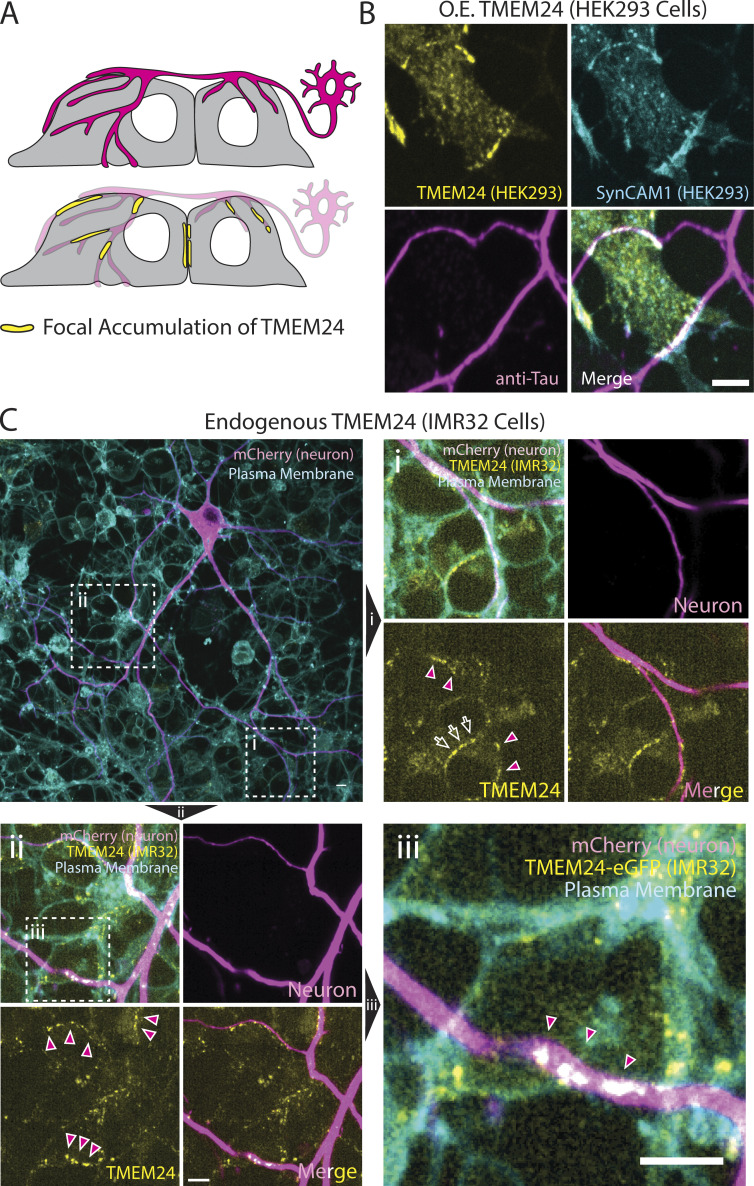
**Coculture system showing that overexpressed TMEM24 in HEK293 cells or endogenous TMEM24 in IMR32 cells localizes at contacts with rat hippocampal neurons. (A)** Diagram of the coculture system showing TMEM24 accumulated beneath neuronal processes. **(B)** TMEM24-mCherry and SynCAM1(563)-eGFP co-expressed in HEK293 cells accumulate at contacts with axons of co-plated rat hippocampal neurons as revealed by anti-tau immunofluorescence. **(C)** Endogenous TMEM24 (endo-eGFP) in IMR32 cells accumulates at contacts with the neuronal processes of a coplated hippocampal neuron expressing mCherry-CAAX (magenta). PMs of the field were labeled with CellBrite 650. **(Ci–Ciii)** show higher magnification of the boxed regions. Note that neurons are only sparsely transfected with mCherry-CAAX so that at least some linear arrays of endo-eGFP spots not in register with mCherry-labeled axons may correspond to unlabeled axons. Magenta-filled arrowheads point to TMEM24-positive ER/PM contacts that are aligned with the mCherry neuron fluorescence. Open arrows point to TMEM24-positive ER/PM contacts between adjacent IMR32 cells. Scale bars = 5 μm.

SynCAM 1 has been extensively studied in the nervous system. In co-cultures of HEK293 cells with rat hippocampal neurons, overexpression of SynCAM 1 in the HEK293 cells drives functional presynapse formation at the sites where axonal processes of hippocampal neurons contact them (Biederer et al., 2002; Fogel et al., 2007). We performed coculture experiments of hippocampal neurons with HEK293 cells expressing TMEM24-mCh in addition to SynCAM 1(363)-eGFP (see Fig. 9 A for diagram). In these co-cultures, we observed strand-like TMEM24-positive ER/PM junctions at sites where neuronal processes made contact with HEK293 cells, as assessed by anti-Tau immunofluorescence (Fig. 9 B). We also co-plated neuroblastoma IMR32 cells expressing TMEM24 tagged with GFP at the endogenous locus with rat hippocampal neurons expressing the PM marker mCh-CAAX. Also in this system, we found an enrichment of endogenous TMEM24 under neuronal processes (Fig. 9 C and Fig. S5 C), showing that such localization occurs at physiological levels of expression of the proteins involved.

## Discussion

The present study identifies a mechanism that couples cell–cell adhesion to the formation of ER/PM junctions with lipid transport properties. Our studies of TMEM24 suggest that this protein and its close paralogue C2CD2 are key players in this coupling. They do so through a mechanism that is distinct from the previously described property of both proteins to tether the ER to the PM via the binding of their C-terminal polybasic motif to the acidic cytosolic leaflet of the PM.

The accumulation of TMEM24 and C2CD2 at cell–cell interfaces is mediated by the interaction between an evolutionarily conserved small β-sheet motif within the predominantly disordered C-terminal region of TMEM24 and a β-sheet module in the CTD of the protein band 4.1 family. Based on AlphaFold multimer predictions, these two β-sheets interlock with each other to form a single continuous chimeric β-sheet. We confirmed this prediction by showing that a partial deletion of the β-sheet of TMEM24 or the mutation of a critical amino acid (I536) in such β-sheet abolished the interaction. Band 4.1 proteins bind a variety of plasma membrane proteins through their FERM domain. Thus, they can function as bridges between the ER and the PM. Importantly, as several interactors of band 4.1 proteins are cell adhesion molecules, the interaction of TMEM24 with band 4.1 provides an explanation for the concentration of TMEM24 at cell–cell contacts. Accordingly, our study shows that in adjacent HEK293 cells, tagged-TMEM24, band 4.1, and SynCAM 1 co-cluster at symmetric ER/PM junctions. Interestingly, homotypic interactions between HEK293 cells mediated by complexes comprising band 4.1 proteins and SynCAM 1, which also comprise members of the MPP protein family, have been described, all of which were hits in our screen for TMEM24 neighbors at sites of cell adhesion. However, it is quite possible that other cell adhesion proteins that bind band 4.1 besides SynCAM 1, (e.g., CD44, CADM4, β-integrins, and others) may contribute to the localization of protein 4.1, and thus TMEM24, at sites of cell–cell contacts. For example, in our cocultures of neurons with cells expressing tagged endogenous or exogenous TMEM24, cell adhesion proteins other than SynCAM 1, but that bind protein 4.1, may have contributed to the formation of ER/PM junctions at sites of cell adhesion.

As the presence of acidic phospholipids on the cytosolic leaflet is a general feature of the entire PM, the interaction of TMEM24 with protein 4.1 (via the β-sheet motif) and with the acid bilayer (via the polybasic motif) may synergize at sites of cell–cell contacts. The charge-based interactions of the polybasic motif may help strengthen the interaction of TMEM24 and C2CD2 with the PM mediated by 4.1 proteins and do so in a regulated way in the case of TMEM24.

The presence of ER/PM junctions at specialized cell-adhesion sites has been observed in a variety of contexts in different tissues, implying the occurrence of mechanisms to coordinate extracellular interactions with the focal recruitment of the ER at these sites. For example, ER/PM junctions are present at cell–cell junctions along the basolateral surface of epithelial cells (Chung et al., 2022). Symmetrically arranged ER/PM junctions have been described at homotypic cell–cell contacts, including contacts between neuronal somata in the nervous system (Wu et al., 2017). Striking examples of asymmetric junctions where ER/PM cisterns are present in only one of the two participating cellular elements are neuronal synapses where an ER cistern with a narrow lumen is closely apposed to the entire post-synaptic membrane such as at synapses of C-fibers onto motor neurons and between efferent axons and hair cells of the vestibular system (Deardorff et al., 2014; Smith and Sjöstrand, 1961). TMEM24 and C2CD2 could cooperate with other tethers to form some of these structures, as other mechanisms have also been described to generate ER/PM junctions at sites of cell adhesion. For example, the Kv2.1 potassium channel, which mediates the formation ER/PM junctions by interacting with VAP, binds the cell adhesion PM protein AMIGO (Maverick et al., 2021; Peltola et al., 2011). There is vast literature on Kv2.1-positive ER/PM junctions being localized to areas of cell–cell contact, both at GABAergic synapses and at contact sites between neurons and astrocytes or microglia where the channel is thought to modulate neuronal activity, synaptic transmission, and response to ischemic insults (Cserép et al., 2020; Du et al., 1998; Misonou et al., 2008; Panzera et al., 2022).

The specific feature of TMEM24 and C2CD2 containing ER/PM junctions, however, is the presence of a lipid transport module in these proteins, implying that lipid transport is a process that occurs at these sites. Cell–cell contacts have an important role in intercellular signaling. Many of the membrane-bound ligands and receptors that mediate these cell–cell interactions trigger intracellular signaling cascades, which include phosphorylation of PI(4,5)P_2_ to PI(3,4,5)P_3_, PLC-mediated PI(4,5)P_2_ cleavage, and other lipid metabolic reactions (Balla, 2013; Di Paolo and De Camilli, 2006). Thus, it is plausible that TMEM24 and C2CD2 may participate in homeostatic responses to lipid perturbations mediated by such reactions. In this context, it is of interest that at least some of the synapses mentioned above with a postsynaptic ER cistern, for example, C-fibers synapses, are cholinergic with the presence postsynaptically of phospholipase C-coupled muscarinic receptors (Deardorff et al., 2014). Thus, cleavage of PI(4,5)P_2_ triggered by acetylcholine release at these sites to generate IP3 and other intracellular second messengers requires lipid exchanges between the PM and the ER to replenish the depleted PI(4,5)P_2_ pool (Chang and Liou, 2015; Kim et al., 2015; Yadav et al., 2015), a process in which TMEM24 and C2CD2 may participate (Lees et al., 2017; Xie et al., 2022).

Finally, proteins of the 4.1 family bind a plethora of cell surface proteins beyond proteins specialized for cell adhesion. Examples of such ligands include AMPA receptors, NMDA receptors, mu-opioid receptors, and metabotropic glutamate receptors. Thus, although our present findings have emphasized an action of TMEM24 at sites of cell adhesion, the interaction of TMEM24 and C2CD2 with proteins of the band 4.1 family may function in ER/PM cross-talk in a variety of additional contexts.

## Materials and methods

### Plasmids

TMEM24-eGFP, TMEM24-mCherry, TMEM24(1–414)-eGFP, TMEM24(1–630)-eGFP, TMEM24(1–666)-eGFP, TMEM24(5S→E)-eGFP, and C2CD2-eGFP constructs were previously reported (Lees et al., 2017; Sun et al., 2019). Briefly, coding sequences were amplified using human cDNA and then truncated or mutated using site-directed mutagenesis, restriction enzyme-based cloning, and other standard laboratory cloning techniques. Kv2.1-eGFP, Kv2.1loopBAD, and hBirA were kind gifts from Dr. M. Tamkun at Colorado State University. GFP-ESyt2 was described previously (Giordano et al., 2013). Briefly, the cDNA of human E-Syt2 was subcloned into the pEGFP-N1 vector. YFP-STIM1(D76A) was a gift from Dr. G. Voeltz (#186622; Addgene). M1R was a gift from Dr. B. Hille. TRAPγ was a kind gift from Dr. M. Mariappan at Yale University. TRAPγ-GFP was subsequently created by cloning TRAPγ into the EGFP-N1 vector using XhoI and HindIII cut sites. TRAPγ-TMEM24(1–414)-eGFP was generated using the TRAPγ-GFP and TMEM24(1–414)-GFP plasmids. TMEM24(Δβ + 5S → E)-eGFP was generated using site-directed mutagenesis to remove a portion of the C-terminus of TMEM24(5S → E)-eGFP including the first three β-strands of the β-sheet band 4.1 interacting domain (Quik-Change II XL; Agilent Technologies). eGFP-Sec61β-APEX2 was cloned using eGFP-Sec61β and eGFP-APEX2 and XhoI and XmaI cut sites. TMEM24-APEX2-eGFP was cloned using Tom20-APEX2-eGFP and TMEM24-eGFP and XhoI and XmaI cut sites. TMEM24(1–414)-APEX2-GFP, TMEM24(1–630)-APEX2-GFP, and TMEM24(1–666)-APEX2-GFP were generated from TMEM(1–414)-GFP, TMEM24(1–630)-GFP, and TMEM24(1–666)-GFP, respectively, using TMEM24-APEX2-eGFP and standard restriction enzyme cloning techniques. mCherry-4.1G, mCherry-4.1G(ΔCTD), and Mem-mCherry-4.1G(CTD) were generous gifts from the I. Cheeseman lab (plasmids #46361, #46360, and #46362, respectively; Addgene). eGFP-4.1R was generated by using pTK81_GFP-4.1R (again a gift from I. Cheeseman, #46352; Addgene) and cloning into CMV-eGFP-C1 vector using XhoI and SacII cut sites. The TMEM24 β-strand point mutations were generated de novo by Epoch Life Sciences based on a series of provided sequence maps and verified by sequencing. SynCAM 1(363)-GFP has been previously described (Fogel et al., 2007). Briefly, eGFP was inserted using a double BmgBI/EcoRV restriction enzyme digest and blunt ligation.

### Cell culture and transfection

HEK293 cells were cultured at 37°C and 5% CO_2_ in DMEM (cat 11965-092; Gibco) + 10% FBS. IMR32 neuroblastoma cells with TMEM24 endogenously tagged with GFP were accomplished via CRISPR/Cas9 by the company PNAbio and were previously described (Sun et al., 2019). IMR32 cells were cultured at 37°C and 5% CO_2_ in DMEM (cat. 11965-092; Gibco), 15% FBS, 1 mM sodium pyruvate (cat. 11360-070; Gibco), and 2 mM glutaMAX (cat 35050-061; Gibco). For differentiation, IMR32 cells were cultured in media supplemented with 2.5 µM bromo-deoxyuridine. The differentiation medium was replaced by 50% every 2–3 days and cells were used for experiments after 14–21 days of differentiation when clear neuronal-like processes could be seen by simple light microscopy. HEK293 cells, IMR32 cells, and differentiated IMR32 cells were transfected using lipofectamine 2000 (cat. 52887; Invitrogen) and used for experiments the following day. For HEK293-HEK293 co-culture experiments, populations of cells were transfected separately, allowed to express the protein of interest overnight, and then plated together on Mattek dished the following morning. Microscopy was performed later that afternoon/evening after the cells had several hours to settle and adhere to the new dish. All cells were checked for mycoplasma contamination monthly. For HEK293-rat hippocampal co-culture experiments, we used a previously published protocol (Biederer and Scheiffele, 2007). Briefly, rat hippocampal neurons were collected from postnatal day 0 or 1 animals and grown in culture until days in vitro 7–10. Simultaneously, HEK293 cells were transfected with the protein of interest (TMEM24-mCherry, mCherry-JPH4, and/or SynCAM 1(363)-GFP; see individual experiment for construct details) and seeded atop the neuronal culture the following day. 24–48 h after combining the cultures, the dishes were fixed in DPBS + 4% formaldehyde for 15 min. Cell membranes were permeabilized with 0.5% CHAPS in DPBS and blocked before being labeled with either rabbit anti-synaptophysin (101 002; Synaptic Systems) or mouse anti-tau (4019S; Cell Signaling) primary antibodies and either donkey anti-rabbit AlexaFluor 647 (A31573; Invitrogen) or goat anti-mouse AlexaFluor 680 (A21058; Invitrogen) secondary antibodies. All work with rats was performed in accordance with both Yale institutional and federal guidelines.

### Microscopy

Imaging was performed using an Andor Dragonfly spinning-disk confocal imaging system with a Zyla CMOS camera and 60× plan apochromat objective (63×, 1.4 NA, oil). For live cell experiments, cell media was replaced with HEPES-buffered live-cell imaging solution (cat. A14291DJ; Invitrogen), and imaging was performed at 37°C and 5% CO_2_. Labeling with Cellbrite and Halo dyes and wash steps were done in imaging saline immediately prior to imaging. CellBrite (cat. 30108; Biotium) was used at 1:1,000 for 7–10 min before washing with imaging saline. Janelia JF646 HaloTag was used at 1:1,000 for 10 min before wash.

### Oxotremorine-M treatment

For the Oxotremorine-M dissociation of TMEM24, cells were transfected with TMEM24-mCh, M1R(unlabeled) and stained using a far-red CellBrite 650 PM dye. During image acquisition, after a baseline was established, Oxo-M dissolved in imaging saline was added to the dish to a final concentration of 10 μM while TMEM24 localization was recorded.

### Image processing and analysis

All microscopy image processing was performed using ImageJ software. Images were pseudocolored, cropped, and adjusted for contrast and brightness. Background subtraction, filtering, and noise removal, where necessary, were performed equally across all channels to generate clearer pictures for publication. Analysis was performed on either unprocessed images or images that had undergone equal background subtraction across channels but no further processing. For normalization, averages of pretreatment values were used as a baseline.

### Quantification of protein enrichment at cell-adjacent regions (enrichment ratio)

The “enrichment ratio” used throughout the manuscript was determined by measuring the mean fluorescence intensity (after background subtraction) for regions of a cell plasma membrane that were in contact with a neighboring cell or facing empty space and dividing those values by the mean measured fluorescence of the non-adjacent region.

$$Enrichment\;Ratio=\frac{\left[Mean\;Measured\;Fluorescence\;of\;Indicated\;Region\right]}{\left[Mean\;Measured\;Fluorescence\;of\;Non\;Adjacent\;Region\right]}$$

This method results in normalization to 1 for all non-adjacent regions and values higher or lower than 1 for the cell-adjacent regions if the protein of interest is enriched or excluded. Cell adjacent and cell non-adjacent regions were determined using CellBrite Steady 650 plasma membrane dye. For these experiments, only cell regions that were adjacent to untransfected cells were used, as fluorescence from the neighboring cells would confound the measurement. In the case of endogenous TMEM24-eGFP IMR32 cells, the population of cells was homogenous, and thus all adjacent regions would include fluorescence from both opposed cells. To compensate for this problem, measurements at cell adjacent regions were divided by half before calculating the enrichment ratio. Outliers were removed if they were more than three standard deviations away from the mean.

### APEX2 proteomic analysis

Our APEX2 protocol is based on the published protocol of the Ting laboratory (Hung et al., 2016). Briefly, HEK293 cells were transfected with the indicated plasmids and allowed to express the proteins over a 24-h period. Cells were then incubated with 500 μM biotin tyramide (cat. 41994-02-9; Iris Biotech) in DMEM + 10% FBS for 1 h, treated with 1 mM H_2_O_2_ for 1 min to induce the APEX reaction, treated with quenching solution (10 mM sodium ascorbate, 5 mM Trolox and 10 mM sodium azide, in DPBS) to end radical formation, washed 3× in PBS and either fixed in DPBS + 4% formaldehyde for 15 min (if the cells were to be imaged) or collected in DPBS + protease inhibitor (for affinity purification). Cells that were imaged were labeled using CF640-conjugated streptavidin to label biotinylated proteins (CF640R Streptavidin, cat 29041; Biotium). Affinity purification was performed using streptavidin-conjugated magnetic beads (REF 88817; Pierce) utilizing the steps and buffers of the previously referenced protocol (Hung et al., 2016). The affinity-purified samples were then either submitted for mass spectrometry analysis or subjected to gel electrophoresis to generate the western blot figures found in the manuscript. Membranes were probed with mouse anti-GFP (G6539; Sigma-Aldrich) then labeled with LiCor IRDye 680LT Goat α-Mouse (926-68020) and LiCor IRDye 680RD Streptavidin (926-68079). Membranes were imaged on a LiCor Odyssey Classic system. LC MS/MS with label-free quantitation was performed on purified samples in triplicate after a precipitation step to reduce detergent and free biotin concentration. A threshold of 2+ unique peptides identified was used, and proteins that did not satisfy this requirement were not included in the analysis.

### Mass spectrometry sample preparation

APEX eluted protein solutions were submitted to the Keck MS & Proteomics Resource at the Yale School of Medicine for mass spectrometry analyses. Proteins were extracted utilizing a cold acetone protein precipitation method. Briefly, 400 µl cold acetone (−20°C) was added to the ∼90 µl eluted protein samples and vortexed. The precipitation was allowed to continue overnight in a −20°C freezer; then the mixture was immediately centrifuged at 14.6 K at 4°C for 10 min. The pellet was then air-dried (but not to completeness) and then reconstituted in 20 µl 0.1% Rapigest (Waters Inc.,) containing ammonium bicarbonate (ABC). The proteins were then reduced with 2 µl of 45 mM dithiothreitol (DTT) at 37°C for 30 min and cooled to room temperature. Alkylation was done with 2 µl 100 mM iodoacetamide at room temperature for 30 min in the dark. 2 µl of trypsin (1:5 of a 0.5 µg/µl trypsin in H_2_O) was then added and incubated at 37°C overnight (e.g., 16 h). Digest samples were then quenched (and Rapigest was crashed out) with 1.3 µl 20% TFA at 37°C for 45 min. The supernatant containing the peptides for analyses was then moved to a new Eppendorf tube. An aliquot was taken, concentration was measured via Nanodrop, and then diluted to 0.05 µg/µl with 0.1% TFA. 1:10 dilution of 10X Pierce Retention Time Calibration Mixture (cat. 88321) was added to each sample prior to injecting on the UPLC Q-Exactive Plus to check for retention time variability the normalization during LFQ data analysis.

### Mass spectrometry

Peptides were analyzed by LC–MS/MS using either a Q-Exactive Plus mass spectrometer equipped with a Waters nanoACQUITY ultraperformance liquid chromatography (UPLC) system using a Waters Symmetry C18 180 mm by 20 mm trap column and a 1.7 mm (75 mm inner diameter by 250 mm) nanoACQUITY UPLC column (35°C) for peptide separation. Trapping was done at 5 µl/min, 99% Buffer A (100% water, 0.1% formic acid) for 3 min. Peptide separation was performed at 300 nl/min with a linear gradient that would reach 5% Buffer B (100% CH_3_CN, 0.075% formic acid) at 2 min, 25% B at 140 min, and 40% B at 165 min, and 90% B at 170 min for 10 min; then dropped down to 3% B at 182 min for 5 min. For the LCMS/MS data-dependent acquisition on the Q-Exactive Plus mass spectrometer, high-energy collisional dissociation (HCD) MS/MS spectra were filtered by dynamic exclusion (20 s) and acquired for the top 20 peaks with charge states 2–6 with m/z isolation window of 1.7. All MS (Profile) and MS/MS (centroid) peaks were detected in the Orbitrap.

### MS data analysis

Mass spectral data were processed using Progenesis QI (v.4.2; Waters Inc.). The analysis method is described elsewhere by Torregrossa et al. (2019). Briefly, peaks were picked in Progenesis QI, and LC MS/MS mascot generic file (.mgf) was exported for protein search with in-house MASCOT Search engine. Protein searches were conducted against the *Homo sapiens* SWISSProt protein database using Mascot Search Engine (v. 2.6.0; Matrix Science; LLC). Mascot search parameters included: parent peptide ion tolerance of 10.0 ppm, peptide fragment ion mass tolerance of 0.020 Da, strict trypsin fragments (enzyme cleavage after the C terminus of K or R, but not if it is followed by P), variable modification of phospho (S, T, Y, and H), oxidation (M), and carboxyamidomethyl (C).

### Co-immunoisolation

HEK293 cells were transfected with the indicated proteins and allowed to express the proteins overnight. Cells were washed twice with DPBS before being lysed in ice-cold IP Lysis/Wash Buffer (Cat. 88804; Pierce; 0.025 M Tris, 0.15 M NaCl, 0.001 M EDTA 1% NP40, and 5% glycerol, pH 7.4). The lysis reaction was allowed to proceed for 5 min. Then cells were centrifuged at 13,000 *g* for 10 min and the supernatant was collected. A portion of this supernatant was set aside as the “input” condition. Then RFP-Trap magnetic agarose beads (Cat. rtma10; ChromoTek) prewashed with IP lysis/wash buffer were added to the cell supernatant and allowed to incubate in a circular rotator overnight at 4°C. Beads were isolated using a magnetic rack and washed with lysis/wash buffer twice before boiling in Laemmli buffer for 10 min. Samples were subjected to gel electrophoresis and membranes were probed with rabbit anti-GFP (cat. Ab290; Sigma-Aldrich) and mouse anti-mCherry 1C51 (cat. Ab125096; Abcam) primary antibodies overnight at 4°C. For secondary antibodies, IRDye 680LT goat anti-mouse (cat. 926-68020; LiCor) and IRDye 800 CW donkey anti-rabbit (cat. 926-32213; LiCor) were used. Membranes were imaged on a LiCor Odyssey Classic system.

### Online supplemental material

Fig. S1 depicts spinning disk microscopy images of ER/PM tethers and their location on the plasma membrane with respect to adjacent cells as quantified in Fig. 1 of the manuscript. Fig. S2 depicts ER/PM contacts in adjacent coplated cells formed by various tethers as well as the localization of these tethers when coexpressed with TMEM24 in a single cell. Fig. S3 shows the localization of the TMEM24(414–630) fragment and its sequence conservation across species. Fig. S4 depicts a western blot of the biotinylated protein bands generated using the constructs outlined in Fig. 4 of the manuscript. Fig. S5 contains spinning disk microscopy images depicting colocalization between TMEM24 and band 4.1R, between C2CD2 and band 4.1G, and between endogenous TMEM24 in IMR32 cells and coplated rat hippocampal neuronal processes. Table S1 shows proteins identified in the APEX2 screen.

## Supplementary Material

Table S1 shows proteins identified in the APEX2 screen.

SourceData F5 is the source file for Fig. 5.

SourceData F7 is the source file for Fig. 7.

SourceData FS4 is the source file for Fig. S4.

## Data Availability

All data are available in the published article and its online supplemental material. The mass spectrometry proteomics data underlying Fig. 4 have been deposited to the ProteomeXchange Consortium via the PRIDE (Perez-Riverol et al., 2022) partner repository with the dataset identifier PXD047112.
