# Supplementary figures and images for: A complex of the lipid transport ER proteins TMEM24 and C2CD2 with band 4.1 at cell–cell contacts
Source: J Cell Biol. 2024 Aug 19;223(11):e202311137. doi: 10.1083/jcb.202311137 (PMC11334333; doi:10.1083/jcb.202311137)

# Figure 5J

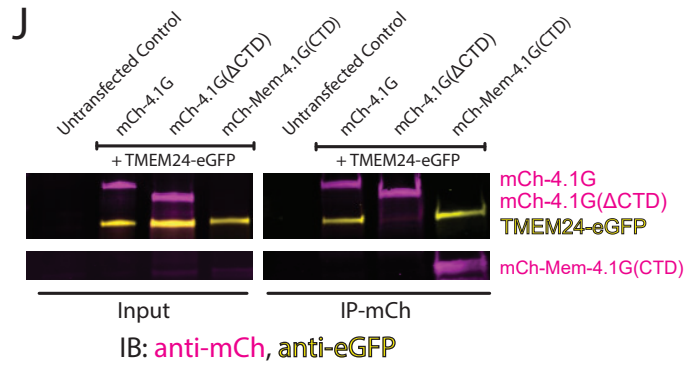

# Source Data

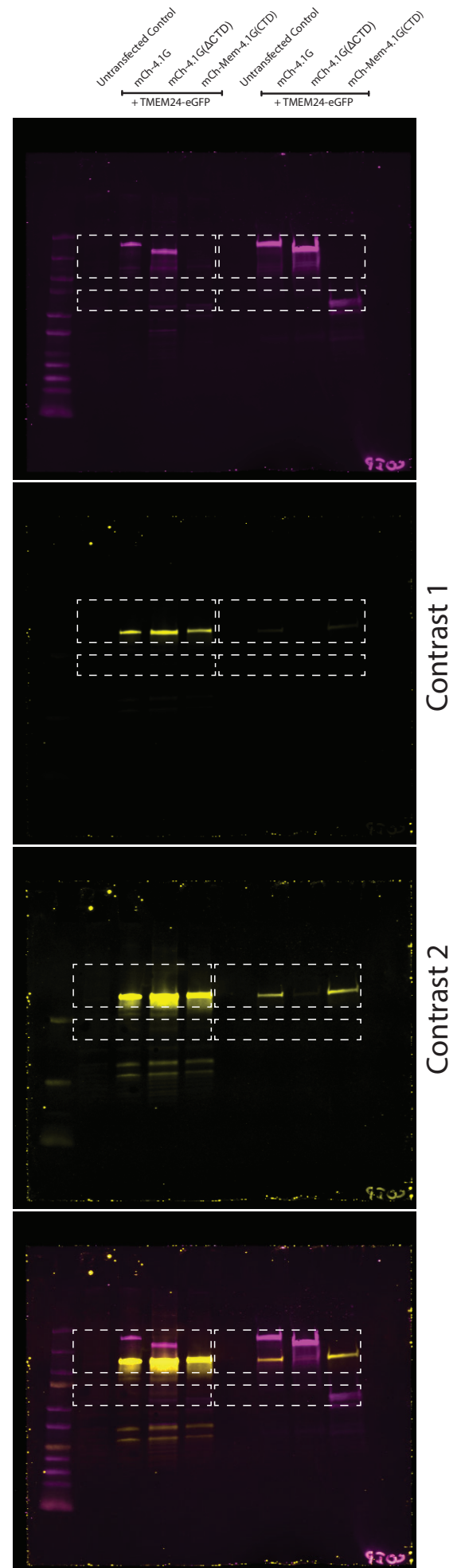

Supplement: SourceData F5 — is the source file for Fig. 5. [file JCB_202311137_SourceDataF5.pdf]

# Figure 7I

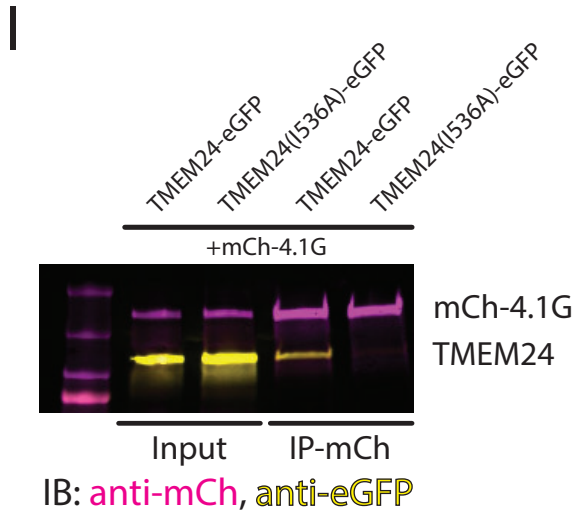

# Source Data

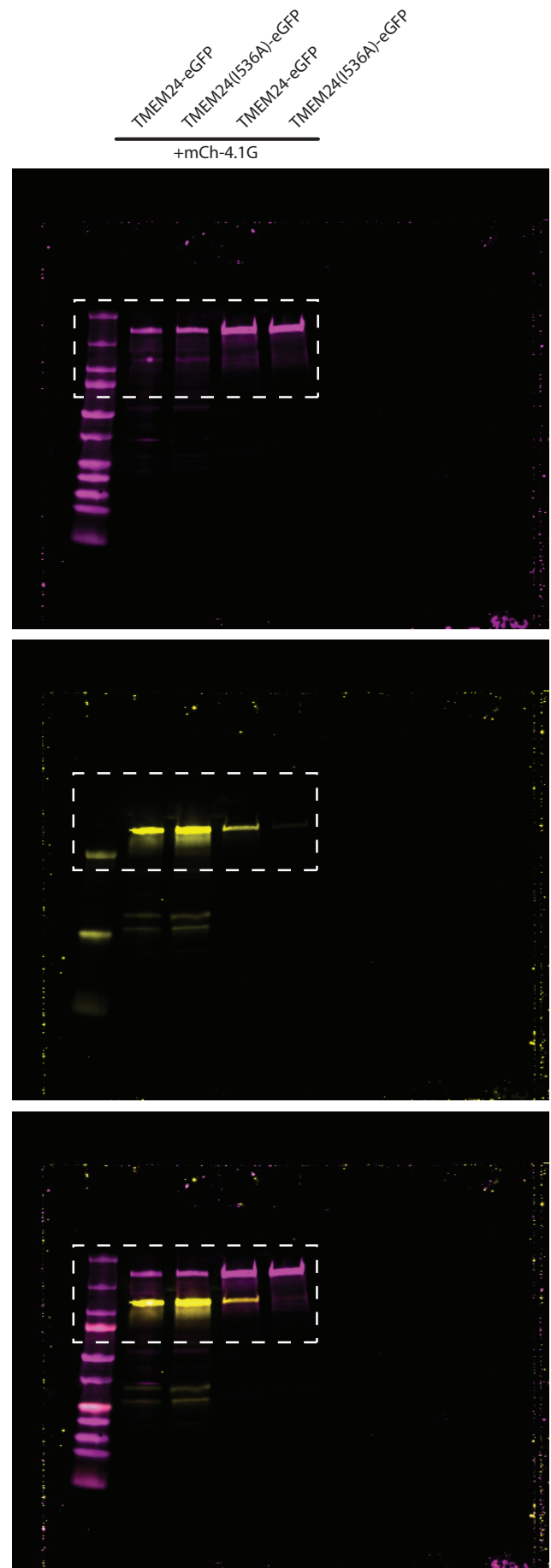

Supplement: SourceData F7 — is the source file for Fig. 7. [file JCB_202311137_SourceDataF7.pdf]

Source Data

Figure S4

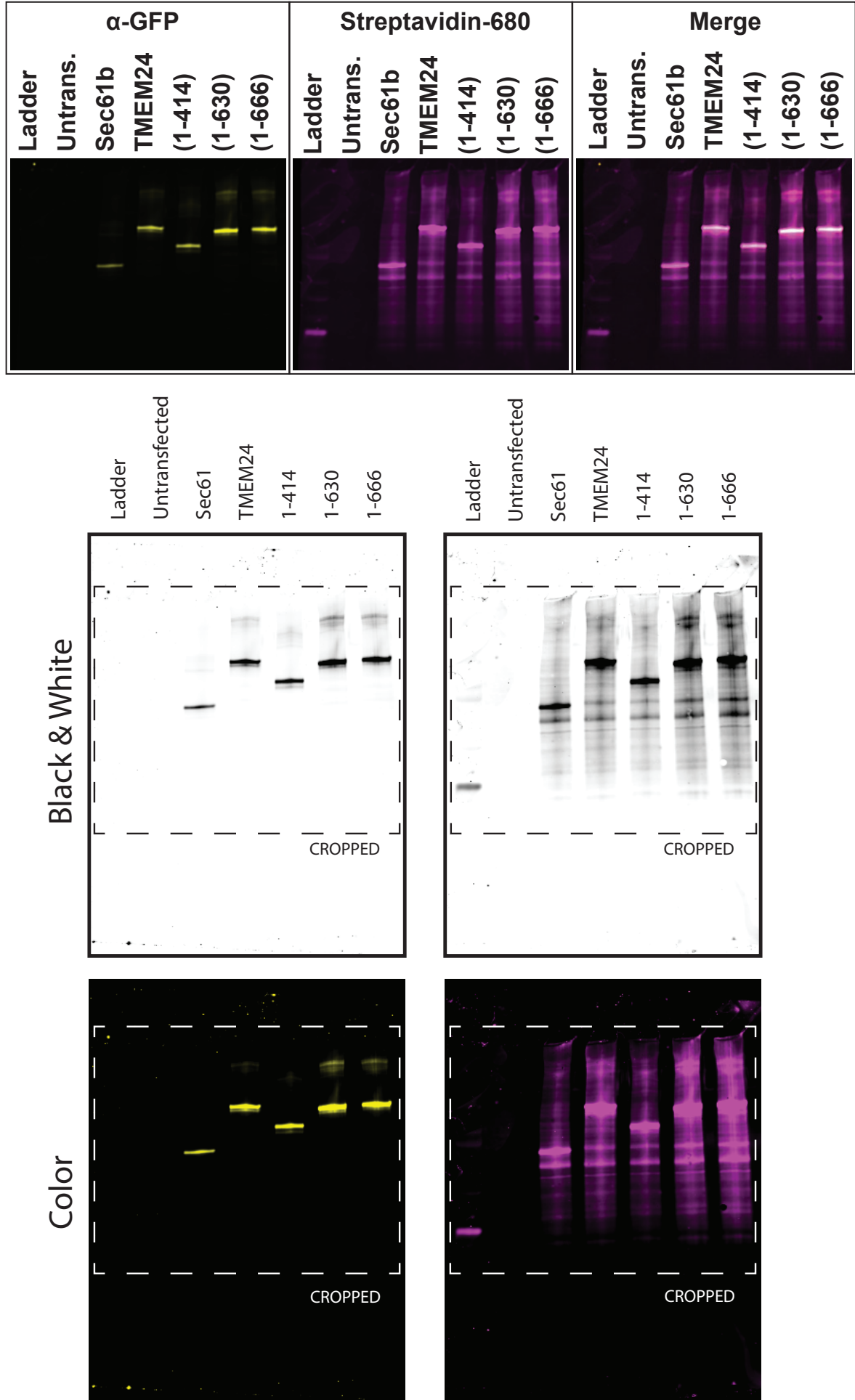

Supplement: SourceData FS4 — is the source file for Fig. S4. [file JCB_202311137_SourceDataFS4.pdf]
